# Cancer-derived exosomal miR-25-3p promotes pre-metastatic niche formation by inducing vascular permeability and angiogenesis

**DOI:** 10.1038/s41467-018-07810-w

**Published:** 2018-12-19

**Authors:** Zhicheng Zeng, Yuling Li, Yangjian Pan, Xiaoliang Lan, Fuyao Song, Jingbo Sun, Kun Zhou, Xiaolong Liu, Xiaoli Ren, Feifei Wang, Jinlong Hu, Xiaohui Zhu, Wei Yang, Wenting Liao, Guoxin Li, Yanqing Ding, Li Liang

**Affiliations:** 1 Department of Pathology, Nanfang Hospital, Southern Medical University, Guangzhou, 510515 Guangdong Province People’s Republic of China; 2 Department of Pathology, Southern Medical University, Guangzhou, 510515 Guangdong Province People’s Republic of China; 3 Guangdong Province Key Laboratory of Molecular Tumor Pathology, Guangzhou, 510515 Guangdong province People’s Republic of China; 4 Department of Pathology, Dongguan People’s hospital, Dongguan, Guangdong Province People’s Republic of China; 5 Department of General Surgery, The Third Affiliated Hospital of Southern Medical University, Guangdong Province, People’s Republic of China; 6 Department of General Surgery, Nanfang Hospital, Southern Medical University, Guangzhou, 510515 Guangdong Province People’s Republic of China

## Abstract

Cancer-derived exosomes are considered a major driver of cancer-induced pre-metastatic niche formation at foreign sites, but the mechanisms remain unclear. Here, we show that miR-25-3p, a metastasis-promoting miRNA of colorectal cancer (CRC), can be transferred from CRC cells to endothelial cells via exosomes. Exosomal miR-25-3p regulates the expression of VEGFR2, ZO-1, occludin and Claudin5 in endothelial cells by targeting KLF2 and KLF4, consequently promotes vascular permeability and angiogenesis. In addition, exosomal miR-25-3p from CRC cells dramatically induces vascular leakiness and enhances CRC metastasis in liver and lung of mice. Moreover, the expression level of miR-25-3p from circulating exosomes is significantly higher in CRC patients with metastasis than those without metastasis. Our work suggests that exosomal miR-25-3p is involved in pre-metastatic niche formation and may be used as a blood-based biomarker for CRC metastasis.

## Abstract (web summary)

The mechanisms underlying pre-metastatic niche formation by cancer derived exosomes is unclear. Here they show that colorectal cancer (CRC) derived exosomal miR-25-3p promotes vascular leakiness and angiogenesis, CRC metastasis, and is upregulated in CRC pateints with metastasis, and suggest miR-25-3p as a biomarker for CRC metastasis.

## Introduction

Formation of pre-metastatic niche provides a supportive microenvironment at foreign sites for disseminating tumor cells, which is characterized by inflammation, immunosuppression, angiogenesis, vascular permeability, lymphangiogenesis, organotropism, and reprogramming^1–4^. Several pre-metastatic niche biomarkers have been found to be helpful for cancer diagnosis and prognosis predication^5,6^. Furthermore, targeting the pre-metastatic niche may be a promising strategy to intervene cancer metastasis^7,8^. Therefore, it’s of great value to identify biomarkers participating in pre-metastatic niche formation that could be used for diagnosis, prognosis, and intervention of cancer metastasis.

Exosomes are small vesicles ranging from 30 to100 nm in size that contain proteins, lipids, as well as various types of nucleic acids, including DNA, RNA, and miRNAs^9^. Exosomes are one of the cancer-derived factors priming pre-metastatic niche formation in distant organs^10–13^. Recently, cancer-secreted exosomes have been reported to be responsible for cancer-induced vascular permeability, inflammation, and bone marrow progenitor cell recruitment in the distant organs, which support engraftment and survival of incoming metastatic cells^14,15^. Most importantly, exosomes derived from the serum of cancer patients have been proved to be reliable markers for cancer diagnosis^16,17^. However, how cancer-derived exosomes regulate pre-metastatic niche formation by inducing angiogenesis and vascular permeability needs to be further investigated.

Herein, we identify that CRC-derived exosomal miR-25-3p can be transferred to vascular endothelial cells and thereby promotes vascular permeability and angiogenesis by targeting Krüppel-like factor 2 (KLF2) and Krüppel-like factor 4 (KLF4). In addition, we demonstrate that exosomal miR-25-3p mediates the formation of a pre-metastatic niche in nude mice by inducing vascular leakiness and consequently promotes CRC metastasis. Finally, our clinical data suggest that miR-25-3p from circulating exosomes of CRC patients may be used as a blood-based biomarker for prediction of metastasis.

## Results

### CRC-secreted miR-25-3p is transferred to endothelial cells

**Fig. 1:**
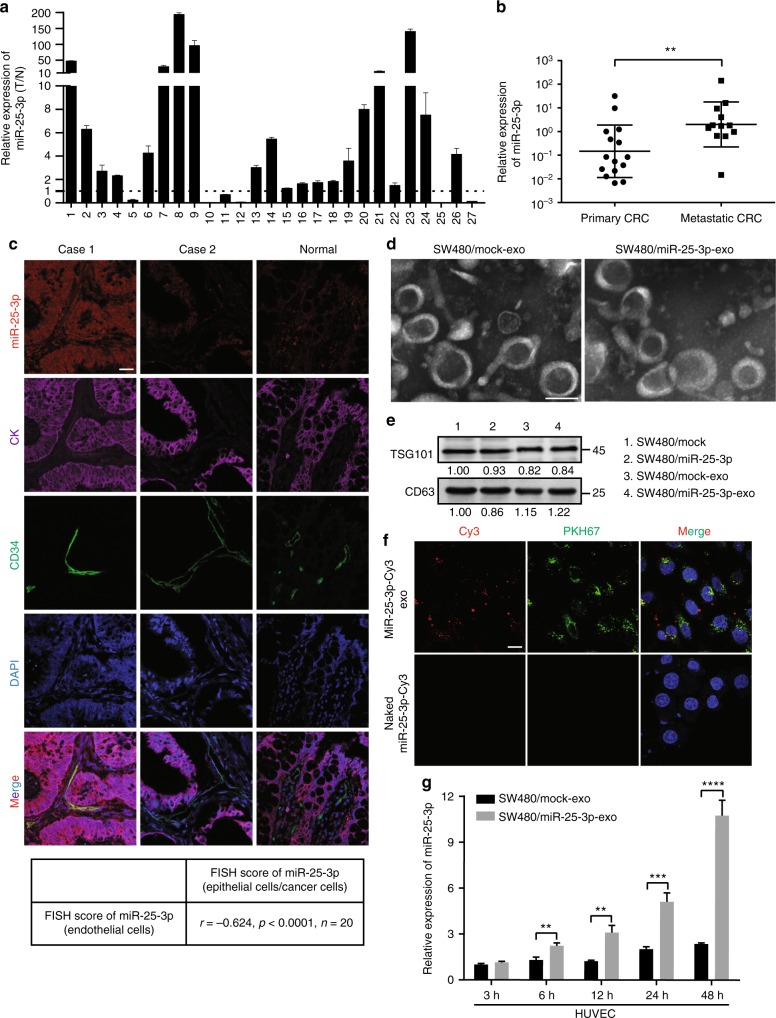
CRC-secreted miR-25-3p is transferred to endothelial cells. **a** RT-PCR analysis of miR-25-3p expression in 27 paired fresh CRC tissues and matched adjacent normal mucosa. The expression of miR-25-3p in normal mucosa was normalized to 1. Mean ±  SEM are provided (*n* = 3). **b** RT-PCR analysis of miR-25-3p expression in 27 cases of the primary CRC tissues with or without metastasis (17 cases without metastasis, 10 cases with metastasis). Mean ± SEM are provided. **c** Correlation analysis of miR-25-3p (red) expression in colorectal epithelial cells/cancer cells (labeled by CK, purple) and their adjacent endothelial cells (labeled by CD34, green). MiR-25-3p levels were determined by FISH and scored as described in Methods. Pearson’s correlation coefficient (r) and *P-*value are shown (*n* = 20). *P-*value is from Spearman’s test. Scale bar represents 50 µm. **d** Transmission electron microscopy of exosomes derived from SW480/mock and SW480/miR-25-3p. Scale bar represents 50 nm. **e** Western blotting analysis of TSG101 and CD63 in SW480/mock, SW480/miR-25-3p and their exosomes. **f** The top panels show presence of Cy3 fluorescence and PKH67 lipid dye in HUVECs after adding PKH67-labeled exosomes derived from SW480 cells for 48 h. HUVECs incubated with naked-miR-25-3p-Cy3 were used as a negative control (the bottom panels). Scale bar represent 10 µm. **g** RT-PCR analysis of miR-25-3p expression in HUVECs incubated with exosomes derived from SW480/mock and SW480/miR-25-3p for 3 h, 6 h, 12 h, 24 h, and 48 h. Mean ± SEM are provided (*n* = 3). **P < 0.01, ***P < 0.001, ****P < 0.0001 according to two-tailed Student’s *t* test

To identify potential metastasis-associated miRNAs in CRC, miRNA array was performed using CRC tissues with or without metastasis and corresponding normal mucosa. Among the differently expressed miRNAs, six miRNAs (miR-221, miR-92a, miR-92b, miR-25-3p, miR-1246, and miR-371-5p) were significantly upregulated in CRC tissues with metastasis compared with those without metastasis (Supplementary Figure 1A). RT-PCR analyses in 27 paired fresh CRC tissues showed that among the six candidate miRNAs, miR-221, miR-92a, miR-25-3p, and miR-1246 were markedly upregulated in CRC tissues (Fig. 1a, Supplementary Figure 1B–F). Most importantly, miR-25-3p level was closely related to metastasis (Fig. 1b, Supplementary Figure 1G–I). In addition, miR-25-3p was obviously upregulated in five CRC cell lines compared with NCM460, a normal human colon mucosal epithelial cell line (Supplementary Figure 2A). FISH analysis showed that miR-25-3p was expressed in both CRC epithelium and stroma, which was consistent with the previous study^18^. Interestingly, the level of miR-25-3p (red) in CRC cells (labeled by CK, purple) positively correlated with that in cancer-adjacent endothelial cells (labeled by CD34, green, Fig. 1c).

It was shown that miRNA can be transferred from cells to cells via exosomes^19^. Thus, we hypothesized whether miR-25-3p could be transferred from CRC cells to endothelial cells via exosomes. To address this question, miR-25-3p was overexpressed or knockdown in CRC cells (Supplementary Figure 2B). Exosomes extracted from condition media of SW480/miR-25-3p and control cells were identified as membrane-encapsulated particles with a range of 50 to 100 nm in size by transmission electron microscopy (Fig. 1d). Additionally, western blot revealed that the vesicles were positive for exosome markers CD63 and TSG101 (Fig. 1e). RT-PCR showed that over-expression or knockdown of miR-25-3p in CRC cells led to the upregulation or downregulation of exosomal miR-25-3p, respectively (Supplementary Figure 2B-C). Besides, miR-93 and miR-106b, two other clustered miRNAs of the miR-25-3p, didn’t compensate for miR-25-3p knockdown in CRC cells (Supplementary Figure 2D and E). Next, HUVECs were incubated with PKH67-labeled exosomes derived from SW480 cells that were transfected with Cy3-labeled miR-25-3p mimics. Both Cy3 fluorescence and PKH67 lipid dye were observed in the incubated HUVECs (Fig. 1f). These results suggest that miR-25-3p is contained in cancer-secreted exosomes and can be transferred to HUVECs. We further detected the levels of miR-25-3p and pri-miR-25-3p in HUVECs incubated with exosomes derived from SW480/mock and SW480/miR-25-3p cells. MiR-25-3p, but not pri-miR-25-3p, was markedly up-regulated in HUVECs incubated with SW480/miR-25-3p exosomes (Fig. 1H, Supplementary Figure 2F). However, miR-25-3p was not upregulated in SW480 incubated with HUVEC/miR-25-3p exosomes (Supplementary Figure 2G). Moreover, with the treatment of Annexin V, an inhibitor of exosome internalization^20^, SW480/miR-25-3p exosomes failed to induce increased miR-25-3p in HUVECs (Supplementary Figure 2H). These results indicate that cancer-secreted miR-25-3p can be transferred to HUVECs through exosomes.

### MiR-25-3p induces vascular leakiness and angiogenesis

**Fig. 2:**
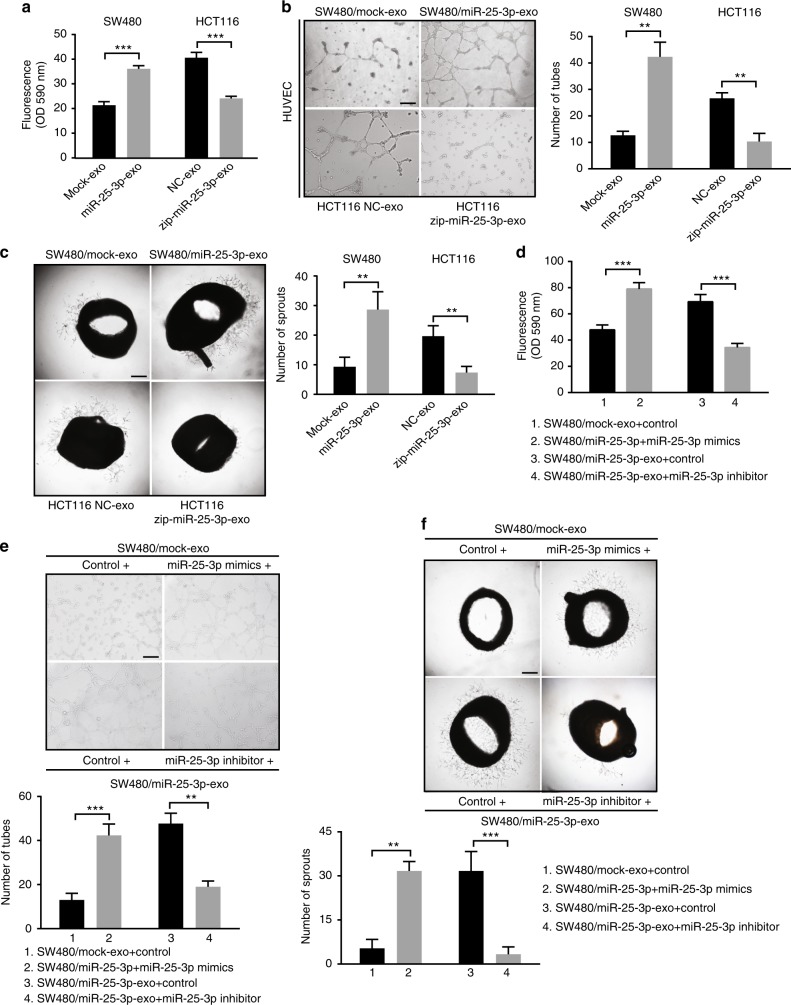
MiR-25-3p induces vascular leakiness and angiogenesis. **a** Permeability of the HUVEC monolayers to rhodamine–dextran (70 kDa) after exposure to exosomes derived from SW480/mock, SW480/miR-25-3p, HCT116 NC, and HCT116/zip-miR-25-3p for 72 h. Mean ± SEM are provided (*n* = 3). **b** Effect of exosomes derived from SW480/mock, SW480/miR-25-3p, HCT116 NC, and HCT116/zip-miR-25-3p on tube formation ability of HUVECs by tube formation assay. Mean ± SEM are provided (*n* = 3). Scale bar represents 100 µm. **c** Effect of exosomes derived from SW480/mock, SW480/miR-25-3p, HCT116 NC, and HCT116/zip-miR-25-3p on vascular outgrowth of rat aortic rings. Vascular outgrowth was quantified by counting all sprouts from one ring. Mean ± SEM are provided (*n* = 3). Scale bar represents 200 µm. **d** Effect of SW480/mock exosomes loading with miR-25-3p mimics and SW480/miR-25-3p exosomes loading with miR-25-3p inhibitor on permeability of HUVEC monolayers. Mean ± SEM are provided (*n* = 3). **e** Effect of SW480/mock exosomes loading with miR-25-3p mimics and SW480/miR-25-3p exosomes loading with miR-25-3p inhibitor on tube formation ability of HUVECs. Scale bar represents 100 µm. Mean ± SEM are provided (*n* = 3). **f** Effect of SW480/mock exosomes loading with miR-25-3p mimics and SW480/miR-25-3p exosomes loading with miR-25-3p inhibitor on vascular outgrowth of rat aortic rings. Scale bar represents 200 µm. Mean ± SEM are provided (*n* = 3). **P < 0.01, ***P < 0.001 according to two-tailed Student’s *t* test

To explore the impact of exosomal miR-25-3p on endothelial cells, we detected whether SW480/miR-25-3p exosomes promote the migration of HUVECs. As was shown in Supplementary Figure 3A, treatment with SW480/miR-25-3p exosomes dramatically promoted migration of HUVECs. In contrast, treatment with HCT116/zip-miR-25-3p exosomes significantly decreased the migration of HUVECs. However, treatment with HUVEC/miR-25-3p exosomes had no effect on the migration of CRC cells (Supplementary Figure 2B, C). Tube formation assay, aortic ring assay, and in vitro permeability assay were then performed to detect whether exosomal miR-25-3p regulates vascular permeability and angiogenesis. Compared with control, exosomes from SW480/miR-25-3p or NCM460/miR-25-3p obviously promoted vascular permeability and angiogenesis. On the contrary, HCT116/zip-miR-25-3p exosomes dampened vascular permeability and angiogenesis (Fig. 2a–c, Supplementary Figure 3D, E). Furthermore, exosomal RNA from SW480/miR-25-3p cells, but not from SW480/mock cells, significantly promoted vascular permeability and angiogenesis (Supplementary Figure 3F, G). To determine whether exosomal miR-25-3p is involved in vascular leakiness and angiogenesis, miR-25-3p mimics were transfected into SW480/mock exosomes to enhance the function of miR-25-3p, while miR-25-3p inhibitor was used to blocked the function of miR-25-3p in SW480/miR-25-3p exosomes. The results showed that exosomal miR-25-3p-induced vascular permeability and angiogenesis were abolished by miR-25-3p inhibitor. Besides, SW480/mock exosomes that were transfected with miR-25-3p mimics significantly promoted vascular permeability and angiogenesis (Fig. 2d, e). These data suggest that exosomal miR-25-3p derived from CRC cells disrupts the integrity of endothelial barriers and induces angiogenesis.

### KLF2 and KLF4 are functional targets of miR-25-3p in HUVECs

**Fig. 3:**
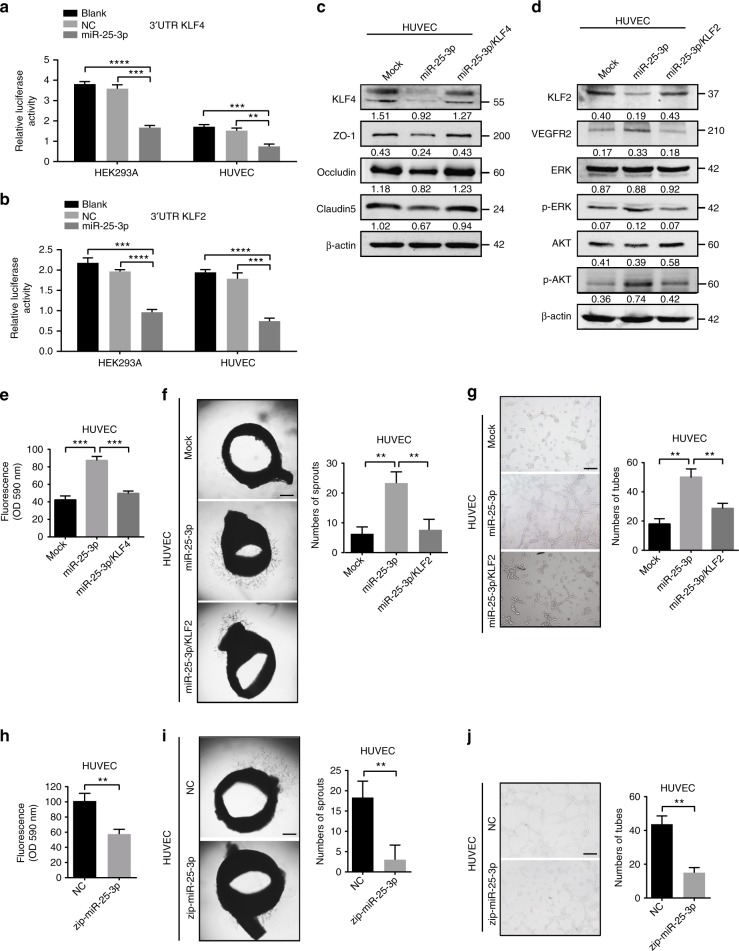
KLF2 and KLF4 are functional targets of miR-25-3p in HUVECs. **a**, **b** Luciferase activities of 3’UTR KLF2-luc and 3’UTR KLF4-luc constructs in HEK293A, HUVECs after transfection of miR-25-3p mimics. Mean ± SEM are provided (*n* = 3). **c** KLF4, ZO-1, occludin, Claudin5 expression in miR-25-3p overexpressing or miR-25-3p/KLF4 co-expressing HUVECs by western blot. **d** KLF2, VEGFR2, AKT, p-AKT, ERK, p-ERK expression in miR-25-3p overexpressing or miR-25-3p/KLF2 co-expressing HUVECs by western blot. **e** Effects of miR-25-3p and miR-25-3p/KLF4 on permeability of HUVEC monolayers by in vitro permeability assay. Mean ± SEM are provided (*n* = 3). **f** Effects of miR-25-3p and miR-25-3p/KLF2 on vascular outgrowth of rat aortic rings. Scale bar represents 200 µm. Mean ± SEM are provided (*n* = 3). **g** Effects of miR-25-3p and miR-25-3p/KLF2 on tube formation ability of HUVECs by tube formation assay. Scale bar represents 100 µm. Mean ± SEM are provided (*n* = 3). **h** Effect of miR-25-3p knockdown on permeability of HUVEC monolayers by in vitro permeability assay. **i** Effect of miR-25-3p knockdown on vascular outgrowth of rat aortic rings. Scale bar represents 200 µm. Mean ± SEM are provided (*n* = 3). **j** Effect of miR-25-3p knockdown on tube formation ability of HUVECs by tube formation assay. Scale bar represents 100 µm. Mean ± SEM are provided (*n* = 3). **P < 0.01, ***P < 0.001 according to two-tailed Student’s *t* test

To explore how miR-25-3p could regulate vascular permeability and angiogenesis, four mRNAtarget-predicting algorithms (miRanda, miRDB, miRWalk, and Targetscan) were utilized to identify the potential downstream targets of miR-25-3p. Among the potential targets, KLF2 and KLF4 were overlapped among all databases. It has been reported that KLF2 dampens angiogenesis by inhibiting promoter activity of VEGFR2^21^. KLF4 is supposed to be crucial for integrity of endothelial barrier because it enhances the promoter activities of tight junction related proteins, including ZO-1, occludin, and Claudin5^22^. To evaluate the role of KLF2 and KLF4 in vascular permeability and angiogenesis, in vitro permeability assay and tube formation assay were performed. Consistent with the previous studies, KLF2 dramatically dampened angiogenesis while KLF4 markedly hampered vascular permeability (Supplementary Figure 4A–F). To establish whether KLF2 and KLF4 are targets of miR-25-3p, 3′ UTRs of KLF2 and KLF4 were cloned into the luciferase plasmid psiCHEK^TM^-2 in HEK293A and HUVECs. Notably, the luciferase activities of 3′ UTR of KLF2 and KLF4 were suppressed by miR-25-3p (Fig. 3a, b). Moreover, ectopic expression of miR-25-3p in HUVECs suppressed the expression of KLF2 and KLF4, while re-introduction of KLF2 or KLF4 plasmid lacking the 3′ UTR region restored the levels of KLF2 or KLF4. Conversely, knocking down of miR-25-3p resulted in upregulation of KLF2 and KLF4. VEGFR2, ZO-1, occludin, and Claudin5 are the downstream genes of KLF2 and KLF4. Overexpression of miR-25-3p in HUVECs increased the level of VEGFR2 and decreased the levels of ZO-1, occludin, and Claudin5, while restoration of KLF2 or KLF4 expression abrogated these effects (Fig. 3c, d, Supplementary Figure 4G). Furthermore, overexpression of KLF2 or KLF4 abolished miR-25-3p-induced vascular permeability and angiogenesis (Fig. 3e–g). However, knockdown of miR-25-3p dampened vascular leakiness and tube formation ability of HUVECs (Fig. 3h–j). These results indicate that miR-25-3p regulates vascular permeability and angiogenesis by silencing KLF2 and KLF4 in HUVECs.

### CRC-secreted miR-25-3p silences KLF2 and KLF4 in HUVECs

**Fig. 4:**
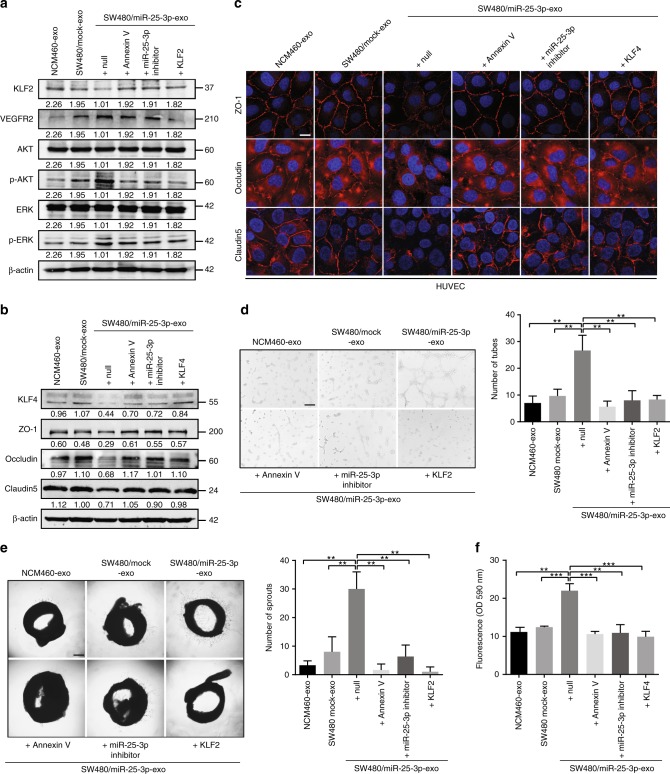
CRC-secreted miR-25-3p silences KLF2 and KLF4 in HUVECs. **a** Western blot analysis of KLF2, VEGFR2, AKT, p-AKT, ERK, p-ERK expression in HUVECs incubated with NCM460 exosomes, SW480/mock exosomes, SW480/miR-25-3p exosomes, SW480/miR-25-3p exosomes + Annexin V, SW480/miR-25-3p exosomes + miR-25-3p inhibitor and SW480/miR-25-3p exosomes + KLF2 groups. **b** Western blot analysis of KLF4, ZO-1, occludin, Claudin5 expression in HUVECs incubated with NCM460 exosomes, SW480/mock exosomes, SW480/miR-25-3p exosomes, SW480/miR-25-3p exosomes + Annexin V, SW480/miR-25-3p exosomes + miR-25-3p inhibitor and SW480/miR-25-3p exosomes + KLF4 groups. **c** Immunofluorescence staining analysis of ZO-1, occludin, Claudin5 expression in HUVECs incubated with NCM460 exosomes, SW480/mock exosomes, SW480/miR-25-3p exosomes, SW480/miR-25-3p exosomes + Annexin V, SW480/miR-25-3p exosomes + miR-25-3p inhibitor and SW480/miR-25-3p exosomes + KLF4 groups. Scale bars represents 10 µm. **d** Effects of NCM460 exosomes, SW480/mock exosomes, SW480/miR-25-3p exosomes, SW480/miR-25-3p exosomes + Annexin V, SW480/miR-25-3p exosomes + miR-25-3p inhibitor and SW480/miR-25-3p exosomes + KLF4 treatments on tube formation ability of HUVECs by tube formation assay. Scale bar represents 100 µm. Mean ± SEM are provided (*n* = 3). **e** Effects of NCM460 exosomes, SW480/mock exosomes, SW480/miR-25-3p exosomes, SW480/miR-25-3p exosomes + Annexin V, SW480/miR-25-3p exosomes + miR-25-3p inhibitor and SW480/miR-25-3p exosomes + KLF2 treatments on vascular outgrowth of rat aortic rings. Scale bar represents 200 µm. Mean ± SEM are provided (*n* = 3). **f** Effects of NCM460 exosomes, SW480/mock exosomes, SW480/miR-25-3p exosomes, SW480/miR-25-3p exosomes + Annexin V, SW480/miR-25-3p exosomes + miR-25-3p inhibitor and SW480/miR-25-3p exosomes + KLF4 treatments on permeability of HUVEC monolayers by in vitro permeability assay. Mean ± SEM are provided (*n* = 3). **P < 0.01, ***P < 0.001 according to two-tailed Student’s *t* test

Next, we assessed the luciferase activities of the KLF2 and KLF4 3′ UTRs in HUVECs incubated with SW480/miR-25-3p exosomes. Obviously, the luciferase activities of KLF2 and KLF4 3′ UTR were dampened by SW480/miR-25-3p exosomes, but not SW480/mock exosomes, indicating that KLF2 and KLF4 in HUVECs can be silenced by exosomal miR-25-3p derived from CRC cells (Supplementary Figure 5A, B). In addition, SW480/miR-25-3p exosomes dramatically decreased the expression levels of KLF2, KLF4, ZO-1, occludin, and Claudin5 while increased the expression levels of VEGFR2, p-AKT, and p-ERK. In contrast, miR-25-3p inhibitor or Annexin V treatment in SW480/miR-25-3p exosomes abolished these effects. Similarly, restoration the expression of KLF2 or KLF4 in exosomes-treated HUVECs rescued the expression of ZO-1, occludin, Claudin5, VEGFR2, p-AKT, and p-ERK (Fig. 4a–c). On the contrary, HUVECs incubated with HCT116/zip-miR-25-3p exosomes had adverse effects (Supplementary Figure 5C). In vitro permeability assay, tube formation assay, and aortic ring assay showed that transfection of SW480/miR-25-3p exosomes with miR-25-3p inhibitor or pretreatment SW480/miR-25-3p exosomes with Annexin V alleviated their function in promoting vascular permeability and angiogenesis. Additionally, KLF2 or KLF4 restoration in recipient cells suppressed these effects (Fig. 4d–f). These results suggest that exosomal miR-25-3p from CRC cells is sufficient to induce vascular permeability and angiogenesis by targeting KLF2 and KLF4.

### CRC-secreted miR-25-3p primes pre-metastatic niche

**Fig. 5:**
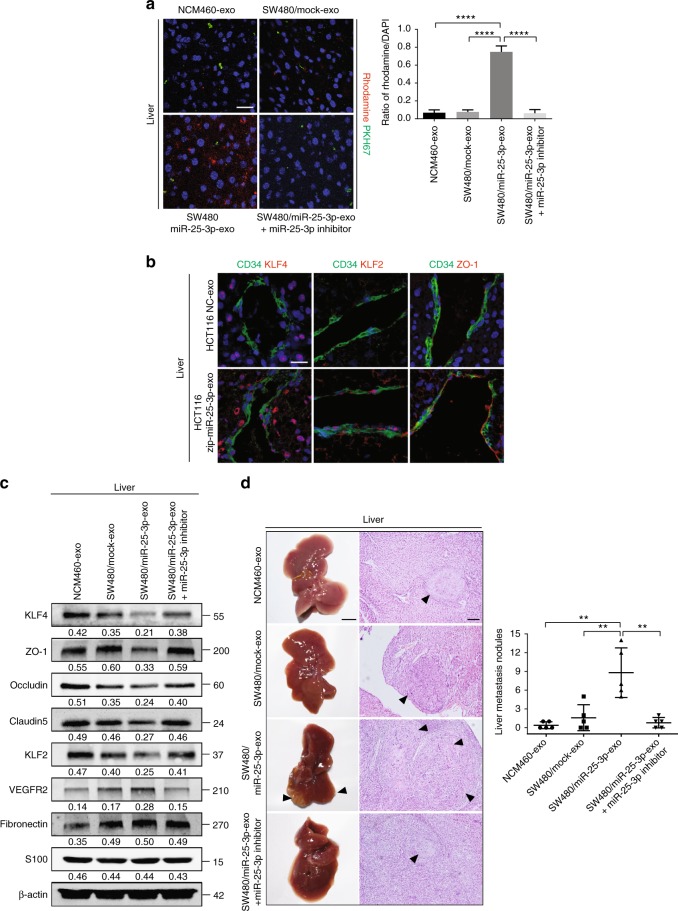
CRC-secreted miR-25-3p primes pre-metastatic niche. **a** Effects of NCM460 exosomes, SW480/mock exosomes, SW480/miR-25-3p exosomes, SW480/miR-25-3p exosomes + miR-25-3p inhibitor treatments on vascular permeability of mice liver by in vivo permeability assay. The mice were injected with rhodamine–dextran after exposure to PKH67-labeled exosomes. Levels of rhodamine–dextran fluorescence in tissues were quantified using Image J software and normalized to the levels of DAPI. Mean ± SEM are provided (*n* = 5). Scale bar represents 50 µm. **b** Effects of NCM460 exosomes, SW480/mock exosomes, SW480/miR-25-3p exosomes, SW480/miR-25-3p exosomes + miR-25-3p inhibitor treatments on vascular KLF4, KLF2, and ZO-1 expression (red) in hepatic vessels by immunofluorescence. The vascular structures were labeled by CD34 (green). Scale bar represents 50 µm. **c** Effects of NCM460 exosomes, SW480/mock exosomes, SW480/miR-25-3p exosomes or SW480/miR-25-3p exosomes + miR-25-3p inhibitor treatments on KLF4, ZO-1, occludin, Claudin5, KLF2, VEGFR2, fibronectin, and S100 expression in mice liver by Western blot. **d** The mice were intra-spleen injected with naked SW480 cells after exposure to NCM460 exosomes, SW480/mock exosomes, SW480/miR-25-3p exosomes or SW480/miR-25-3p exosomes + miR-25-3p inhibitor treatments. The number of liver metastatic sites (indicated by arrows) was counted under the microscope. Mean ± SEM are provided (*n* = 5). Scale bar in left panels represents 0.5 cm. Scale bar in right panels represents 100 µm. **P < 0.01, ****P < 0.0001 according to two-tailed Student’s *t* test

To evaluate whether exosomal miR-25-3p plays a role in vascular permeability in pre-metastatic niche, exosomes from NCM460, SW480/mock, and SW480/miR-25-3p were labeled with PKH67 lipid dye and then injected into tail vein of nude mice. Results of in vivo permeability assay demonstrated that exosomes from SW480/miR-25-3p, but not those from NCM460 and SW480/mock, dramatically promoted vascular permeability in the liver and lung. However, transfection with miR-25-3p inhibitor in SW480/miR-25-3p exosomes abrogated this effect (Fig. 5a, Supplementary Figure 6A). Moreover, injection of HCT116/zip-miR-25-3p exosomes obviously inhibited vascular permeability in the liver and lung (Supplementary Figure 6B). Injection of HCT116/zip-miR-25-3p exosomes resulted in upregulation of KLF2, KLF4, and ZO-1 in the hepatic and pulmonary vessel, compared with HCT116 NC group. (Fig. 5b, Supplementary Figure 6C). Furthermore, western blot showed that injection of SW480/miR-25-3p exosomes resulted in the decreases of KLF4, KLF2, ZO-1, occludin, Claudin5 and an increase of VEGFR2 in the lung and liver, whereas HCT116/zip-miR-25-3p exosomes led to inverse effects (Fig. 5c, Supplementary Figure 7A–C). It has been reported that inflammation, fibronectin deposition, and pericyte coverage played important roles in cancer-induced pre-metastatic niche formation^13,14,23^. Therefore, we evaluated the role of inflammation, fibronectin deposition, and pericyte coverage in the liver and lung from exosomes-treated mice. Obviously, exosomal miR-25-3p had no effect on fibronectin, S100 expression or pericyte coverage in the liver and lung (Fig. 5c, Supplementary Figure 7A–E). To confirm whether miR-25-3p-induced vascular permeability promotes CRC metastasis, SW480 cells were injected into tail veins or spleens of nude mice that were pretreated with exosomes from SW480/miR-25-3p. As expected, there were more hepatic and pulmonary metastatic colonies in mice injected with exosomes from SW480/miR-25-3p than those from NCM460 and SW480/mock. Nevertheless, these effects were rescued after blockade of exosomal miR-25-3p by miR-25-3p inhibitor (Fig. 5d, Supplementary Figure 8A). On the contrary, HCT116/zip-miR-25-3p exosomes dramatically inhibited CRC metastasis, compared with HCT116 NC group (Supplementary Figure 8B, C). Taken together, these results make it clear that cancer-derived exosomal miR-25-3p induces pre-metastatic niche formation, thereby promoting CRC metastasis.

### MiR-25-3p promotes CRC metastasis

**Fig. 6:**
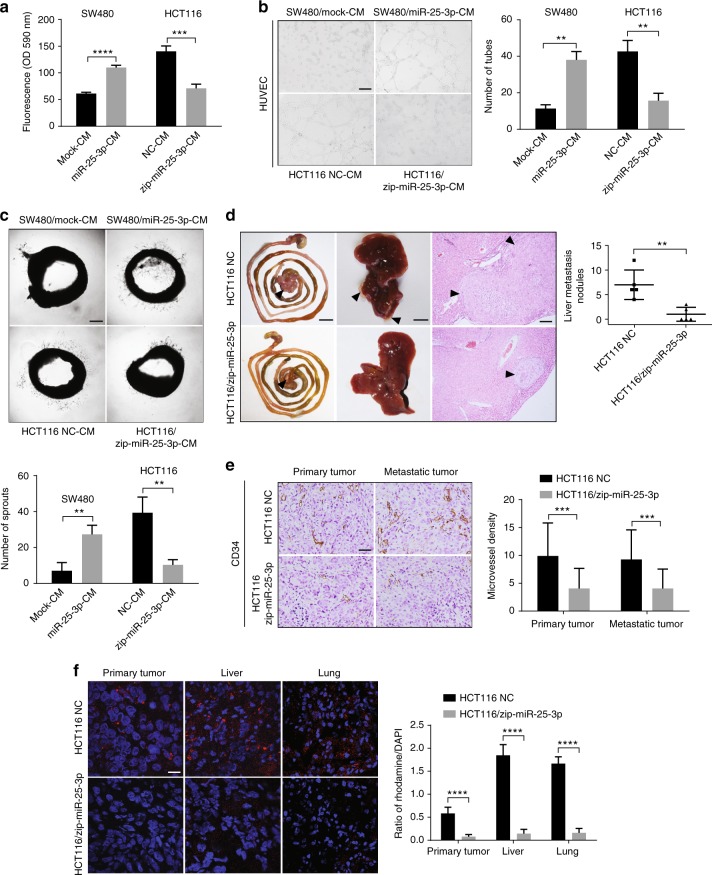
MiR-25-3p promotes CRC metastasis. **a** Effects of condition media derived from SW480/mock, SW480/miR-25-3p and HCT116 NC, HCT116/miR-25-3pon permeability of HUVEC monolayers by in vitro permeability assay. Mean ± SEM are provided (*n* = 3). **b** Top, effects of condition media derived from SW480/mock and SW480/miR-25-3p on tube formation ability of HUVECs by tube formation assay. Bottom, effects of condition media derived from HCT116 NC and HCT116/miR-25-3p on tube formation ability of HUVECs. Scale bar represents 100 µm. Mean ± SEM are provided (*n* = 3). **c** Top, effects of condition media derived from SW480/mock and SW480/miR-25-3p on vascular outgrowth of rat aortic rings. Bottom, effects of condition media derived from HCT116 NC and HCT116/miR-25-3p on vascular outgrowth of rat aortic rings. Scale bar represents 200 µm. Mean ± SEM are provided (*n* = 3). **d** Effect of miR-25-3p knockdown on liver metastasis of mice. HCT116 NC or HCT116/miR-25-3p was injected into the mesentery at the tail end of cecum. The number of lung metastatic sites (indicated by arrows) was counted under the microscope. Mean ± SEM are provided (*n* = 5). Scale bar in left panels represents 1 cm. Scale bar in middle panels represents 0.5 cm. Scale bar in right panels represents 100 µm. **e** Effect of miR-25-3p knockdown on introtumoral microvessel density in primary and metastatic tumor. The number of microvessel was counted under the microscope. Mean ± SEM are provided (*n* = 5). Scale bar represents 50 µm. **f** Effect of miR-25-3p knockdown on hepatic and pulmonary vascular permeability in primary and metastatic tumor. The liver and lung were derived from mice 30 days after being orthotopically implanted with HCT116/zip-miR-25-3p cells. Levels of rhodamine–dextran fluorescence in tissues were quantified using Image J software and normalized to the levels of DAPI. Mean ± SEM are provided (*n* = 5). Scale bar represents 50 µm. **P < 0.01, ***P < 0.001, ****P < 0.0001 according to two-tailed Student’s *t* test

Although it has been reported that miR-25-3p is upregulated in CRC and associated with patient prognosis^24^, the mechanism in which miR-25-3p promotes CRC progression has not been elucidated. Therefore, we asked whether miR-25-3p regulates vascular permeability, angiogenesis, and metastasis in CRC. Conditioned media derived from SW480/miR-25-3p obviously promoted vascular permeability and angiogenesis, while those from HCT116/zip-miR-25-3p dampened vascular permeability and angiogenesis (Fig. 6a–c). To determine the effect of miR-25-3p on CRC metastasis, HCT116 NC or HCT116/zip-miR-25-3p cells were implanted into the tail end of the cecum, and tumor metastasis was monitored. Less hepatic metastases were observed in mice implanted with HCT116/zip-miR-25-3p cells (Fig. 6d). In addition, a lower intratumoral microvessel density was observed in primary tumors and hepatic metastases from HCT116/zip-miR-25-3p group (Fig. 6e), indicating that miR-25-3p modulates cancer-induced angiogenesis. Besides, primary tumors and hepatic metastases from HCT116/zip-miR-25-3p group didn’t show lower expression level of HIFα, suggesting that the angiogenic role of miR-25-3p was independent of HIFα (Supplementary Figure 9A). Furthermore, a dramatically lower vascular permeability was observed in the liver and lung from mice implanted with HCT116/zip-miR-25-3p cells in the pre-metastatic stage (Fig. 6f). Taken together, these data illustrate that miR-25-3p promotes CRC-induces vascular permeability, angiogenesis and contributes to CRC metastasis.

### Serum exosomal miR-25-3p is associated with CRC metastasis

**Fig. 7:**
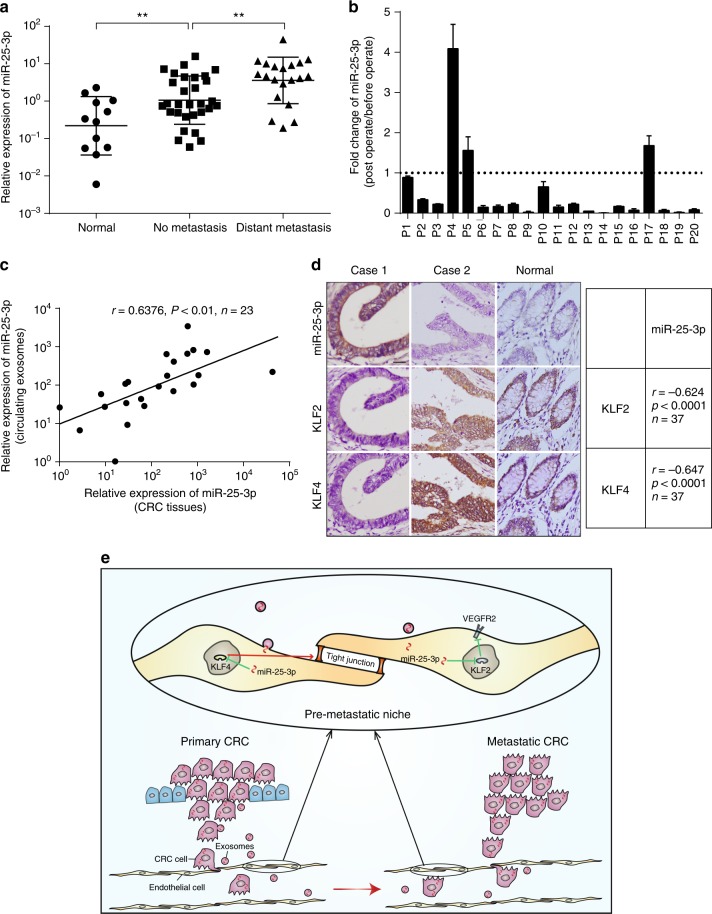
Serum exosomal miR-25-3p is associated with CRC metastasis. **a** RT-PCR analysis of miR-25-3p level in circulating exosomes from healthy donor or CRC patients (12 cases of healthy donors, 36 cases of patients without metastasis, 15 cases of patients with metastasis). **P < 0.01 according to two-tailed Student’s *t* test. **b** Fold change of miR-25-3p in circulating exosomes from 20 CRC patients after surgical resection of CRC tissues. The expression of miR-25-3p in circulating exosomes from CRC patients at post-operation stage was normalized to 1. **c** Spearman correlation analysis between miR-25-3p levels in CRC tissues and miR-25-3p levels in circulating exosomes from CRC patients. Pearson’s correlation coefficient (r) and *P-*value are shown, *n* = 23. *P*-value is from Spearman’s test. **d** Spearman correlation analysis between miR-25-3p expression and KLF2, KLF4 expression in CRC tissues. Pearson’s correlation coefficient (r) and *P-*value are shown. *P-*value is from Spearman’s test. Scale bar represents 50 µm. **e** Schematic diagram of the role of CRC-secreted miR-25-3p in pre-metastatic niche formation

To determine whether the level of exosomal miR-25-3p rises in serum correlated with metastasis, circulating exosomes were  extracted from serum of healthy donors and CRC patients with or without metastasis and then used for the detection of miR-25-3p. RT-PCR analysis showed that miR-25-3p from circulating exosomes was upregulated in CRC patients compared with healthy donors. Moreover, the miR-25-3p levels in circulating exosomes from CRC patients with metastasis were higher than those without metastasis (Fig. 6a). We also detected the expression of miR-25-3p in circulating exosomes of CRC patients before and after operation. In total, 85% (17/20) of patients showed a dramatically decrease of miR-25-3p in circulating exosomes after removal of CRC tissues (Fig. 7b). Furthermore, miR-25-3p expression levels in circulating exosomes and CRC tissues were positively correlated (Fig. 7c), indicating that upregulation of miR-25-3p in CRC tissues may contribute to elevated miR-25-3p level in circulating exosomes. In addition, ISH staining of miR-25-3p and IHC staining of KLF2 and KLF4 in series sections of CRC specimens showed that the expression of miR-25-3p was negatively correlated with KLF2 and KLF4 expression (Fig. 7d). Thus, our clinical data show that a high level of miR-25-3p in circulating exosomes is associated with CRC metastasis.

## Discussion

MiR-25-3p has been reported as a cancer-promoting miRNA that associated with poor prognosis of CRC patients^24–30^. However, the molecular mechanism through which miR-25-3p regulates CRC metastasis has not been delineated. In this study, we unveil that CRC-secreted miR-25-3p can be delivered to vascular endothelial cells via exosomes, then disrupts the integrity of endothelial barriers and induces angiogenesis, consequently contributes to CRC metastasis. Furthermore, we find that the level of serum exosomal miR-25-3p is associated with CRC metastasis. Collectively, our study reveals the role of CRC-secreted miR-25-3p in CRC progression and its clinical significance in CRC.

miRNA can be transferred between cancer cells and stromal cells, which results in efficient silencing of mRNAs to reprogram the target cell transcriptome^19^. For instance, exosomal miR-1247-3p derived from hepatocellular carcinoma cells can be delivered to cancer-associated fibroblast and prime cancer-associated fibroblast activation, consequently promotes hepatocellular carcinoma metastasis^31^. Exosome-mediated miR-19a transferred from astrocytes to tumor cells hampered PTEN expression and aggressive outgrowth in the brain^32^. In summary, these studies suggest that exosomes-mediated transfer of miRNA between cancer cells and stromal cells is essential for cancer progression. Our results of FISH showed that miR-25-3p levels in CRC cells and endothelial cells were positively correlated, based on which we raised the hypothesis that miR-25-3p can be transferred from CRC cells to endothelial cells via exosomes. In addition, we observed Cy3 fluorescence in HUVECs incubated with exosomes from SW480 cells that were transfected with Cy3-labeled miR-25-3p. These results uncovered that CRC-secreted miR-25-3p can be transferred to endothelial cells.

miR-25-3p has been reported to be involved in proliferation^25–28^, apoptosis^29,30^, and motility^25–27^. However, the role of miR-25-3p in vascular endothelial cells has not been elucidated. Cancer-induced vascular permeability and angiogenesis play pivotal roles in cancer metastasis^4^. The current study provides evidences that miR-25-3p in vascular endothelial cells can induce vascular permeability and angiogenesis by downregulating KLF2 and KLF4. Krüppel-like factor (KLF) family, a family of zinc finger-containing transcription factors, regulates many biological processes^33,34^. KLF2 negatively regulates angiogenesis by dampening the promoter activity of VEGFR2^21^, while KLF4 maintains integrity of endothelial barrier function by promoting the promoter activity of tight junction related proteins including ZO-1, occludin, and Caludin5^22^. Furthermore, both KLF2 and KLF4 are downregulated in CRC^35,36^. Our results showed that miR-25-3p in HUVECs downregulated KLF2 and KLF4 and subsequently decreased their downstream targets ZO-1, occludin, Claudin5, and increased the level of VEGFR2. Our data suggest that CRC-secreted exosomal miR-25-3p promotes vascular permeability and angiogenesis by silencing KLF2 and KLF4.

Although several studies verify the essential role of pre-metastatic niche formation in CRC metastasis^37,38^, the mechanisms through which primary CRC cells induce pre-metastatic niche formation are largely unknown. Moreover, multiple studies revealed the emerging role of pre-metastatic niche biomarker in diagnosis, intervention of cancer metastasis, and prognosis predication^6,39,40^. Notably, cancer-induced vascular permeability/angiogenesis is one of the characteristics of pre-metastatic niche. Exosomal miR-105 and 181c from breast cancer destroy vascular endothelial barriers or blood–brain barriers in distant organ during early pre-metastatic stage, which promotes vascular permeability and breast cancer metastasis^6,41^. In the present study, we explored whether exosomal miR-25-3p regulates the pre-metastatic niche formation. In vivo permeability assay showed that exosomal miR-25-3p from CRC cells significantly promoted vascular permeability in mice. Moreover, exosomal miR-25-3p from CRC cells decreased the levels of KLF2, KLF4, ZO-1, occludin, Claudin5, and increased the level of VEGFR2 in the lung and liver of mice. In addition, an increase in metastatic burden was observed in the lung and liver of mice pretreated with SW480/miR-25-3p exosomes. We also found that upregulation of miR-25-3p in CRC cells promoted vascular permeability, angiogenesis, and metastasis. Thus, CRC-secreted miR-25-3p induces the formation of pre-metastatic niche at foreign site by promoting angiogenesis and destroying tight junctions of vein endothelial cells (Fig. 7e). A recent study unveils the critical role of KLF4 in dampening pre-metastatic niche formation and metastasis by modulating perivascular activation^23^. Nevertheless, our data showed that downregulation of KLF4, the target gene of miR-25-3p, resulted in vascular endothelial barrier destruction, which promoted pre-metastatic niche formation. Collectively, we propose a new role for KLF4 in cancer-induced pre-metastatic niche formation and provide new insight into the formation of pre-metastatic niche. Inflammation, fibronectin deposition, and change of pericyte are considered as key characteristics of the pre-metastatic niche^13,14,23^. However, our results suggest that exosomal miR-25-3p modulates neither S100, fibronectin expression nor pericyte coverage in the liver and lung, indicating miR-25-3p-induced pre-metastatic niche formation is independent of inflammation, fibronectin deposition, and pericyte coverage. Cancer therapies targeting pre-metastatic niche are supposed to be a promising strategies to intervene cancer metastasis. Targeting S1PR1-STAT3 signaling axis in myeloid cells inhibits pre-metastatic niche formation and cancer metastasis^39^. Angiopoietin-2 blockade dampens cancer-induced inflammatory and angiogenic response of endothelial cells in the metastatic niche^40^. Our result showed that blockade of exosomal miR-25-3p from CRC cells alleviated hepatic and pulmonary vascular permeability and subsequent CRC metastasis, indicating that miR-25-3p may be used as a therapeutic target to intervene CRC metastasis.

Recent studies reveal the emerging role of exosomal biomarker in cancer diagnosis and prognosis assessment. Exosomal macrophage migration inhibitory factor from pancreatic ductal adenocarcinoma cells (PDAC) contributes to pre-metastatic niche formation in the liver and may be used for diagnosis of PDAC liver metastasis^14^. Exosomal integrins are verified to be potential marker for organ-specific metastasis prediction^15^. Of note, It has been reported that exosomal miR-25-3p from liposarcoma cells promotes liposarcoma progression by inducing the formation of a supportive microenvironment, and it may be used as a circulating marker for prognosis and therapeutic effect of liposarcoma prediction^42^. Here, our data showed that miR-25-3p level in circulating exosomes from CRC patients with metastasis was higher than those without metastasis. In addition, dramatical drop of miR-25-3p level was observed in most of CRC patients who received resection surgery. We also found a positive correlation in miR-25-3p level between circulating exosomes and cancer tissues in CRC patients. Collectively, our clinical data suggest that quantitative blood test for miR-25-3p level in circulating exosomes would be useful for diagnosis of CRC metastasis and patient selection with a high risk of metastasis for preventive treatment.

## Methods

### Microarray analysis

The miRNA expression in normal mucosa (Normal), CRC tissues without metastasis (Non-met), and CRC tissues with distant metastasis (Met) were analyzed using human miRNA Paraflo™ array (LC Sciences, CA, USA). Prior approval was obtained from the Southern Medical University Institutional Board (Guangzhou, China). Informed consent was obtained from each patient on the day of admission. Five clinical samples were pooled in each group. The miRNA array experiment was carried out at Microarray Core Laboratory in LC Sciences (Hangzhou, China).

### Cell lines and human tissue samples

Human umbilical vein endothelial cells (HUVEC), human embryonic kidney 293A cell line (HEK293A), human colon cell line NCM460, human CRC cell lines SW480, LS174T, SW620, LOVO, and HCT116 were purchased from the American Type Culture Collection (ATCC). HEK293A, NCM460, SW480, LS174T, SW620, LOVO, and HCT116 cell lines were cultured in DMEM medium (GIBCO, Gaithersburg, MD, USA) supplemented with 10% fetal bovine serum (HyClone, Logan, USA). HUVECs were cultured in F12-K medium (GIBCO, Gaithersburg, MD, USA) with 10% fetal bovine serum (HyClone, Logan, USA). A total of 57 cases of formalin-fixed paraffin-embedded CRC samples were collected from CRC patients who underwent curative-intent surgery without prior radiotherapy and chemotherapy between 2010 and 2012 at the Department of Pathology, Nanfang Hospital. The 50 freshly collected CRC biopsies and their matched adjacent noncancerous mucosa tissues were collected at the operation room, Nanfang Hospital between 2016 and 2017. The fresh biopsies were frozen and stored in liquid nitrogen until further use. A total of 75 cases of serum samples were collected from CRC patients that underwent curative-intent surgery on the day of surgery and seven days after surgery in Nanfang Hospital between 2016 and 2017. Besides, 12 cases of serum samples were collected from healthy donors. The blood samples were then centrifuged at 2500*g* for 10 min to extract the serum. All the samples were stored at –80 °C until needed. The medical records of the patients were reviewed to collect the information about TNM (T = primary tumor; N = regional lymph nodes; M = distant metastasis) stages. Prior approval was obtained from the Southern Medical University Institutional Board (Guangzhou, China). Informed consent was obtained from each patient on the day of admission.

### RNA interference and plasmids

Inhibitor and mimics of miR-25-3p were purchased from GenePharma. The sequences of inhibitor and miRNA mimics referred above were listed in Supplementary Table 1 and Supplementary Table 2. Lentivirus vectors expressing miR-25-3p and repressing miR-25-3p were constructed and generated by Genechem Inc. In the rescue experiments, cells that stably expressed miR-25-3p or incubated with exosomal miR-25-3p were transfected with the human KLF2 and KLF4 expressing plasmids (GeneCopoeia). For exosomes transfection, miR-25-3p mimics/inhibitor (GenePharma) were loaded in exosomes using Exo-Fect Exosome Transfection Kit (System Biosciences).

### Exosomes isolation, characterization, and treatment

Exosomes were purified from CRC-derived conditioned media or serum of CRC patients by ultracentrifugation. CRC cells were cultured in DMEM medium supplemented with 10% fetal bovine serum. The fetal bovine serum was depleted of exosomes by ultracentrifugation at 110,000×*g* overnight at 4 °C prior to use. Conditioned media were collected after 48 h and centrifuged at 500*g* for 10 min at 4 °C, followed by 16,800*g* for 30 min at 4 °C. The supernatants were passed through a 0.22 um filter (Millipore) and ultracentrifuged at 110,000*g* for 70 min at 4 °C. The exosomes pellets were washed with phosphate-buffered saline (PBS) followed by a second ultracentrifugation at 110,000*g* for 70 min at 4 °C and then resuspended in PBS. The amount of exosomes was measured by the BCA Protein Assay kit (KeyGEN BioTECH). For transmission electron microscopy, exosomes were fixed with 2% paraformaldehyde and placed on 200-mesh Formvar-coated grids. The grids were then stained using 2% phosphotungstic acid for 2 min and observed on a transmission electron microscope (Hitachi H-7500). For exosomes labeling, exosomes were fluorescently labeled using PKH67 membrane dye (Sigma). Labeled exosomes were washed in 10 ml of PBS, collected by ultracentrifugation, and resuspended in PBS. For cell treatment, 2 µg of exosomes were incubated with 2 × 10^5^ recipient cells for 48 h.

### Fluorescence in situ hybridization and immunohistochemistry

Paraffin-embedded tissue blocks were cut into 2.5-μm sections and transferred to glass slides. FISH was performed in tissue sections using fluorescence in situ hybridization (FISH) kit (Bosterbio, USA) and the miR-25-3p detection probe (Biolink, Guangzhou, China) by following the manufacturer’s protocol. For immunohistochemistry (IHC), sections were immersed in 3% hydrogen peroxide to block endogenous peroxidase activity and incubated with primary antibodies overnight at 4 °C. Subsequently, the horseradish-peroxidase-conjugated secondary antibody (DakoCytomation, Glostrup, Denmark) was applied and incubated for 1 h at room temperature. The expression of KLF2, KLF4, and HIFα was visualized by using DAB and counterstained with hematoxylin. The following primary antibodies were used: CD34 (Abcam, ab81289, 1:200 dilution), KLF2 (Abcam, ab203591, 1:100 dilution), KLF4 (Abcam, ab106629, 1:100 dilution), HIFα (Affinity, AF1009, 1:200 dilution).

### Immunofluorescence

Cells grown on glass coverslips or tissue sections were fixed in 4% paraformaldehyde for 10 min at room temperature. Cells were washed twice with PBS. Blocking buffer (DakoCytomation, Glostrup, Denmark) was added for 30 min, and samples were then stained with primary antibodies and goat anti-mouse IgG/Alexa Fluor (Bioss Antibodies, bs-0296G-AF647, bs-0368G-AF488, 1:200 dilution). The following primary antibodies were used: αSMA (Proteintech, 55135-1-AP, 1:200 dilution), CD34 (Abcam, ab81289, 1:200 dilution), KLF2 (Abcam, ab203591, 1:100 dilution), KLF4 (Abcam, ab106629, 1:100 dilution), and Claudin5 (Abcam, ab15106, 1:100 dilution), ZO-1 (Cell Signaling, #8193, 1:1000 dilution), occludin (Abcam, ab216327, 1:100 dilution), CK (Abcam, ab191208, 1:200 dilution).

### RT-PCR

Total RNA was extracted from cells using Trizol reagent (Clontech Laboratories, USA) according to the manufacturer’s instructions. Reverse transcription was performed using Mir-X™ miRNA First-Strand Synthesis Kit for miRNAs (Clontech Laboratories, USA) or PrimeScript™ RT Master Mix for general genes (Clontech Laboratories, USA). Real-time PCR was conducted using SYBR Green PCR Master Mix (Applied TaKaRa, Otsu, Shiga, Japan) and performed on Applied Biosystems 7500 Fast Real-Time RCR System (Applied Biosystems, Foster City, CA, USA). The sequences of all indicated primers were listed in Supplementary Table 3.

### Western blotting

Tissues, cells, and exosomes lysates were prepared in RIPA buffer (KeyGEN BioTECH) and quantified using Bradford Protein Assay (KeyGEN BioTECH). Subsequently, the lysates were subjected to SDS–PAGE, transferred onto PVDF membranes (Millipore). Then membranes were incubated with primary antibodies overnight at 4 °C. The following primary antibodies were used: fibronectin (Santa Cruz, sc-69681, 1:200 dilution), S100 (Santa Cruz, sc-53438, 1:200 dilution), KLF2 (Abcam, ab203591, 1:500 dilution), KLF4 (Abcam, ab106629, 1:1000 dilution) and Claudin5 (Abcam, ab15106, 1:1000 dilution), TSG101 (Abcam, ab125011, 1:1000 dilution), CD63 (Abcam, ab59479, 1:1000 dilution), β-actin (Proteintech, 20536-1-AP, 1:2000 dilution), ZO-1 (Cell Signaling, #8193, 1:1000 dilution), occludin (Abcam, ab216327, 1:1000 dilution), VEGFR2 (Cell Signaling, #9698, 1:200 dilution), ERK (Cell Signaling, #4695, 1:1000 dilution), p-ERK (Cell Signaling, #4370, 1:1000 dilution), AKT (Cell Signaling, #4691, 1:1000 dilution), p-AKT (Cell Signaling, #13038, 1:1000 dilution). Following incubation with the specific HRP-conjugated antibody (Fdbio science, FDM007 or FDR007, 1:10,000 dilution), chemiluminescence signal was detected using FDbio-Femto ECL western blotting detection reagents (Fdbio science, Hangzhou, China). Uncropped scans of the most important blots are shown in Supplementary Figures 10 and 11.

### Migration assay, permeability assay, and angiogenesis assay

For transwell migration assay, the exosomes-treated HUVECs or CRC cells were suspended in serum-free medium and seeded into the transwell chambers with inserts of 8-μm pore size (BD Biosciences). The medium with 10% FBS was placed into the bottom chamber. After 12 h, the cells that had migrated through the membrane and stuck to the lower surface of the membrane were stained with hematoxylin and counted under a light microscope in four random visual fields (200×). Each experiment was repeated three times. For tube formation assay, matrigel matrix (Corning) was plated in 24-well plate and incubated at 37 °C for 30 min to allow the matrigel to polymerize. The treated HUVECs were seeded on the matrigel-coated well. The plate was then incubated at 37 °C in 5% CO_2_ humidified atmosphere. Tube formation was observed at 12 h with microscope. The tube formation ability was determined by measuring the number of tubes. Each experiment was repeated three times. For in vitro permeability assay, rhodamine B isothiocyanate-dextran (average MW ~70,000; Sigma) was added to the top well of the transwell filters (0.4-μm pore size; BD Biosciences) on which treated HUVECs (10^5^ cells per well) were seeded for 3 days. Then medium in the bottom well was collected 30 min later and the appearance of fluorescence was monitored at 544 nm excitation and 590 nm emission^6^. For aortic ring assay, thoracic aortas excised from 8- to 12-week-old Sprague Dawley (SD) rats were cut into 1 mm-long cross-sections. Rings were placed on matrigel-coated wells and incubated at 37 °C for 30 min to allow the matrigel to polymerize. Subsequently, F12-K medium (GIBCO, Gaithersburg, MD, USA) with 10% fetal bovine serum, 50 U/ml penicillin, 50 μg/ml streptomycin, and supplemented with 10 ng/ml vascular endothelial growth factor and 25 μg/ml heparin was added to the well. After 24 h, the aortic rings were incubated with conditioned medium, exosomes derived from CRC cells or transfected with miR-25 mimics, miR-25 inhibitor, or KLF2 overexpressing plasmid. The number of sprouts was observed on day 5 with microscope. Vascular outgrowth was quantified by counting all sprouts from one ring. All assays were performed in triplicate and each experiment was repeated three times.

### Luciferase activity assay

The 3′ UTR segments of the KLF2 and KLF4 genes were amplified by polymerase chain reaction (PCR) and inserted into the vector. Co-transfections of KLF2 and KLF4 3′ UTR plasmids with miR-25-3p lentivirus vector into the cells were accomplished by using Lipofectamine 2000 (Invitrogen). Luciferase activity was measured 48 h after transfection by the Dual-Luciferase Reporter Assay System (Promega). All assays were performed in triplicate and each experiment was repeated three times.

### Animal models

Six-week-old male athymic BALB/c-nu/nu mice were purchased from the Central Laboratory of Animal Science of Southern Medical University (Guangzhou, China), and maintained in a specific pathogen-free environment. All protocols for animal studies were reviewed and approved by the Institutional Animal Care and Use Committee of Southern Medical University. For orthotropic metastasis assay, nude mice were anesthetized and their ceca were exteriorized by laparotomy. Then 2 × 10^6^ CRC cells were injected into the mesentery at the tail end of the cecum. After 60 days, mice were sacrificed and all organs were removed for examination. For education experiments, 6-week-old nude mice were injected with 5 µg of exosomes via tail vein every other day for 2 weeks. Exosomes-treated mice were either killed for tissue collection and assessment, or subjected to the injection of CRC cells or rhodamine–dextran. For in vivo permeability assay, 100 mg/kg rhodamine–dextran was intravenously injected were injected into the tail vein of nude mice 3 h before a transcardiac perfusion was carried out to remove the excess dye. Mice livers and lungs were then removed for examination. For tail vein metastasis assay, 2 × 10^6^ CRC cells were injected into the tail vein of exosomes-treated nude mice. After 30 days, mice injected with CRC cells were sacrificed and lungs were removed for examination. For intra-spleen metastasis assay, nude mice were anesthetized and their spleens were exteriorized by laparotomy. Then 2 × 10^6^ CRC cells were injected into the spleen capsule of exosomes-treated nude mice. After 30 days, mice injected with CRC cells were sacrificed and livers were removed for examination.

### Statistical analysis

Statistical analyses were performed using GraphPad Prism 7 software. Quantitative values of all experiments were expressed as the mean ± SD. Differences among/between sample groups were analyzed by one-way ANOVA or the independent samples *T* test. Pearson’s correlation coefficient was used to measure the degree of the linear relationship between miR-25, KLF2, and KLF4. *P* < 0.05 was considered to be statistically significant. Adobe Illstrator CC, Adobe Photoshop CC, and Image J software were used for figure presentation.

## Supplementary information

Supplementary Information

## Data Availability

The miRNA array data from CRC tissues with or without metastasis and corresponding normal mucosa are deposited at Gene Expression Omnibus (accession number: GSE120300). All other remaining data are included in the article and Supplementary Information files, or available from the authors upon reasonable request.
