# Supplementary material for: Cancer-derived exosomal miR-25-3p promotes pre-metastatic niche formation by inducing vascular permeability and angiogenesis
Source: Nat Commun. 2018 Dec 19;9:5395. doi: 10.1038/s41467-018-07810-w (PMC6300604; doi:10.1038/s41467-018-07810-w)
Supplement: Supplementary file 1 — Supplementary Information [file 41467_2018_7810_MOESM1_ESM.pdf]

Cancer-derived Exosomal MiR-25-3p Promotes Pre-metastatic Niche Formation by Inducing Vascular Permeability and Angiogenesis

Zeng et al.

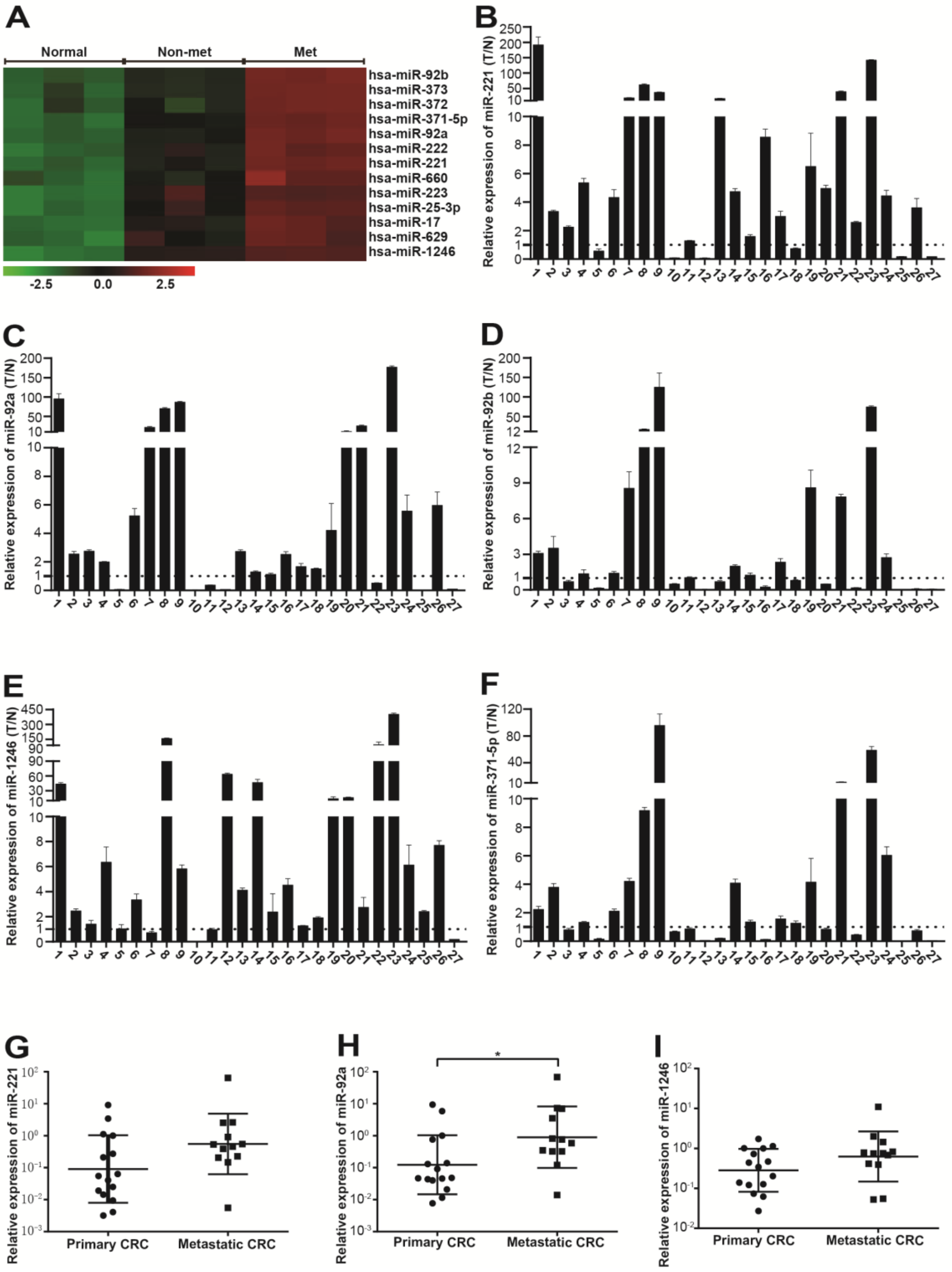

Supplementary Figure 1. MiR-25-3p is a metastasis-associated miRNA in CRC. **(A)** A heatmap representing microarray data for the indicated CRC tissues with and without metastasis and corresponding normal mucosa. Five clinical samples were pooled in each group. **(B-F)** RT-PCR analysis of

miR-221 (B), miR-92a (C), miR-92b (D), miR-1246 (E) and miR-371-5p (F) expression in 27 paired fresh CRC tissues and matched adjacent normal mucosa. The expression of miRNAs in normal mucosa was normalized to 1. Mean ± s.e.m are provided (n=3). **(G-I)** RT-PCR analysis of miR-221 (G), miR-92a (H) and miR-1246 (I) expression in 27 cases of the primary CRC tissues with or without metastasis (17 cases without metastasis, 10 cases with metastasis). Mean ± s.e.m are provided. *P <.05 according to two-tailed Student's t test.

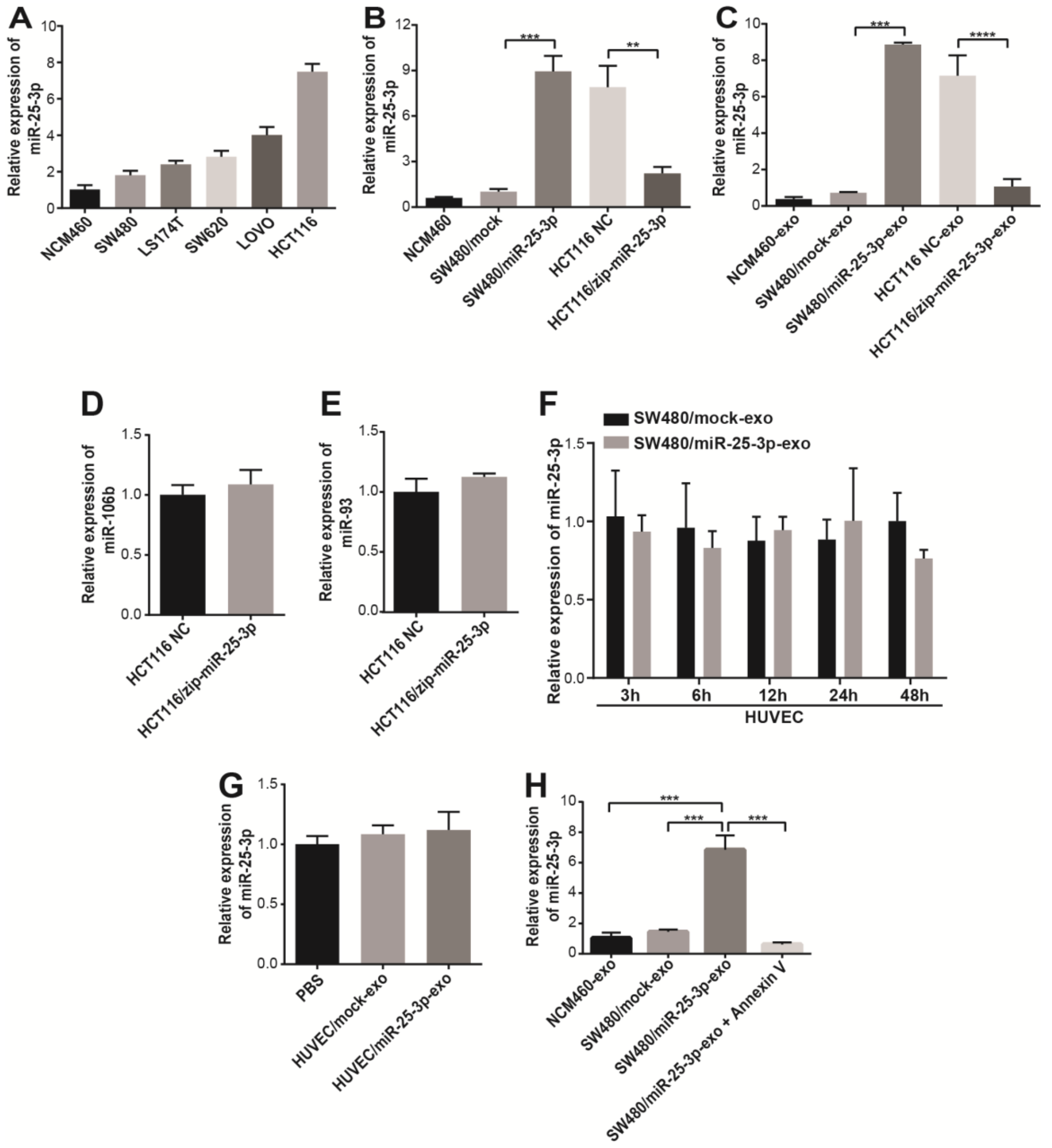

Supplementary Figure 2. CRC-secreted miR-25-3p can be transferred to HUVECs. **(A)** RT-PCR analysis of miR-25 expression in NCM460, SW480, LS174T, SW620, LOVO and HCT116 cell lines. Mean ± s.e.m are provided (n=3). **(B)** RT-PCR analysis of miR-25-3p expression in NCM460, SW480/mock, SW480/miR-25-3p, HCT116 NC and HCT116/zip-miR-25-3p. Mean ± s.e.m are provided (n=3). **(C)** RT-PCR analysis of miR-25-3p

expression in exosomes derived from NCM460, SW480/mock, SW480/miR-25-3p, HCT116 NC and HCT116/zip-miR-25-3p. Mean ± s.e.m are provided (n=3). (**D**) RT-PCR analysis of miR-106b expression in HUVEC NC and HUVEC/zip-miR-25-3p. Mean ± s.e.m are provided (n=3). (**E**) RT-PCR analysis of miR-93 expression in HUVEC NC and HUVEC/zip-miR-25-3p. Mean ± s.e.m are provided (n=3). **(F)** RT-PCR analysis of pri-miR-25-3p expression in HUVECs incubated with exosomes derived from SW480/mock and SW480/miR-25-3p for 3 h, 6 h, 12 h, 24 h and 48 h. Mean ± s.e.m are provided (n=3). (**G**) Effect of PBS, HUVEC/mock exosomes, HUVEC/miR-25-3p exosomes treatments on miR-25-3p expression in SW480. Mean ± s.e.m are provided (n=3). **(H)** Effect of NCM460 exosomes, SW480/mock exosomes, SW480/miR-25-3p exosomes, SW480/miR-25-3p exosomes + Annexin V treatments on miR-25-3p expression in HUVECs. Mean ± s.e.m are provided (n=3). **P<.01, ***P<.001, ****P<.0001 according to two-tailed Student's t test.

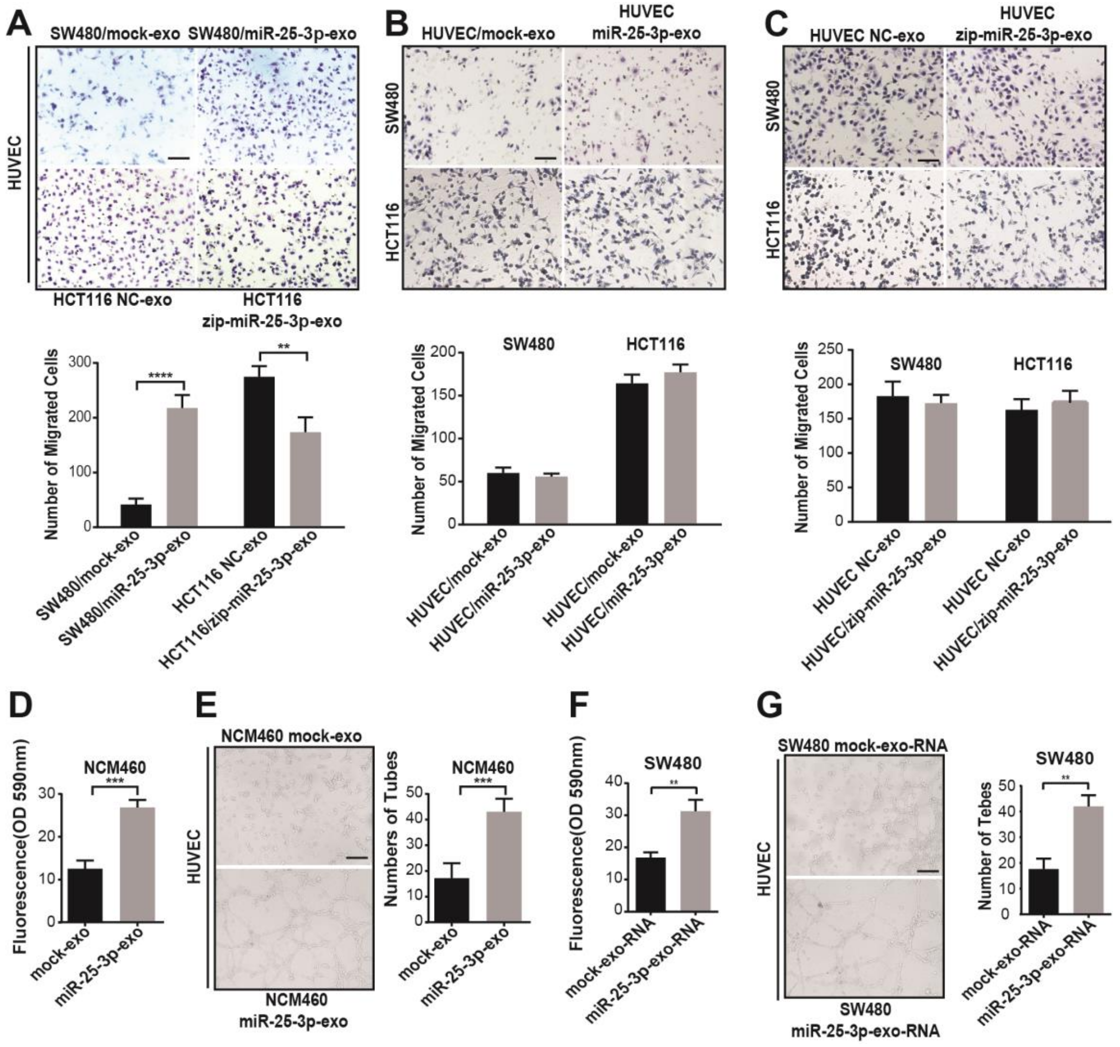

Supplementary Figure 3. MiR-25-3p promoted vascular permeability and angiogenesis. (**A**) Effect of SW480/mock exosomes, SW480/miR-25-3p exosomes, HCT116 NC exosomes and HCT116/miR-25-3p exosomes treatment on migration of HUVECs. Scale bar represents 100 μm. Mean ± s.e.m are provided (n=4). (**B**) Effect of HUVEC/mock exosomes and HUVEC/miR-25-3p exosomes treatment on migration of SW480 and HCT116. Scale bar represents 100 μm. Mean ± s.e.m are provided (n=4). (**C**) Effect of HUVEC NC exosomes and HUVEC/zip-miR-25-3p exosomes treatment on

migration of SW480 and HCT116. Scale bar represents 100 µm. Mean ± s.e.m are provided (n=4). (**D**) Permeability of the HUVEC monolayers to rhodamine-dextran (70 kDa) after exposure to exosomes derived from NCM460/mock, NCM460/miR-25-3p, for 72h. Mean ± s.e.m are provided (n = 3). (**E**) Effect of exosomes derived from NCM460/mock, NCM460/miR-25-3p on tube formation ability of HUVECs by tube formation assay. Mean ± s.e.m are provided (n = 3). Scale bar represents 100 µm. **(F)** Effect of transfecting RNA extracted from SW480/mock exosomes, SW480/miR-25-3p exosomes on permeability of HUVEC monolayers by in vitro permeability assay. Mean ± s.e.m are provided (n = 3). **(G)** Effect of transfecting RNA extracted from SW480/mock exosomes, SW480/miR-25-3p exosomes on tube formation ability of HUVECs by tube formation assay. Mean ± s.e.m are provided (n = 3). Scale bar represents 100 µm. **P<.01, ***P<.001, ****P<.0001 according to two-tailed Student's t test.

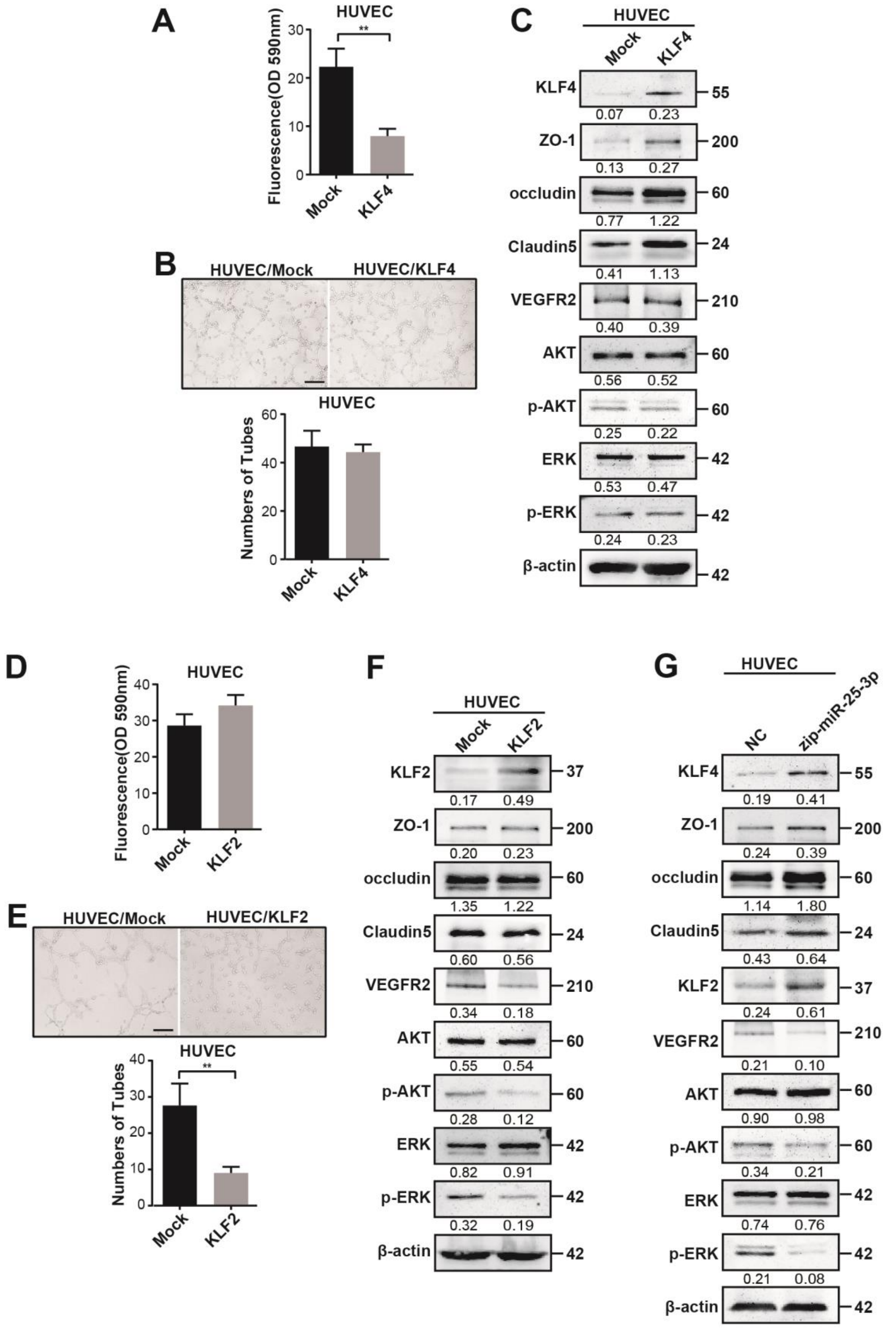

Supplementary Figure 4. The role of KLF2, KLF4 and miR-25-3p in HUVECs.

(A) Effect of KLF4 over-expression on permeability of HUVEC monolayers by in vitro permeability assay. Mean ± s.e.m are provided (n = 3). **(B)** Effect of KLF4 over-expression on tube formation ability of HUVECs by tube formation assay. Mean ± s.e.m are provided (n = 3). Scale bar represents 100 μm. **(C)** KLF4, ZO-1, occludin, claudin 5, VEGFR2, AKT, p-AKT, ERK and p-ERK expression in HUVEC/mock and HUVEC/KLF4 by Western blot. **(D)** Effect of KLF2 over-expression on permeability of HUVEC monolayers by in vitro permeability assay. Mean ± s.e.m are provided (n = 3). **(E)** Effect of KLF2 over-expression on tube formation ability of HUVECs by tube formation assay. Mean ± s.e.m are provided (n = 3). Scale bar represents 100 μm. **(F)** KLF2, ZO-1, occludin, claudin 5, VEGFR2, AKT, p-AKT, ERK and p-ERK expression in HUVEC/mock and HUVEC/KLF2 by Western blot. **(G)** KLF4, ZO-1, occludin, claudin 5, KLF2, VEGFR2, AKT, p-AKT, ERK and p-ERK expression in HUVEC NC and HUVEC/zip-miR-25-3p by Western blot. **P<.01 according to two-tailed Student's t test.

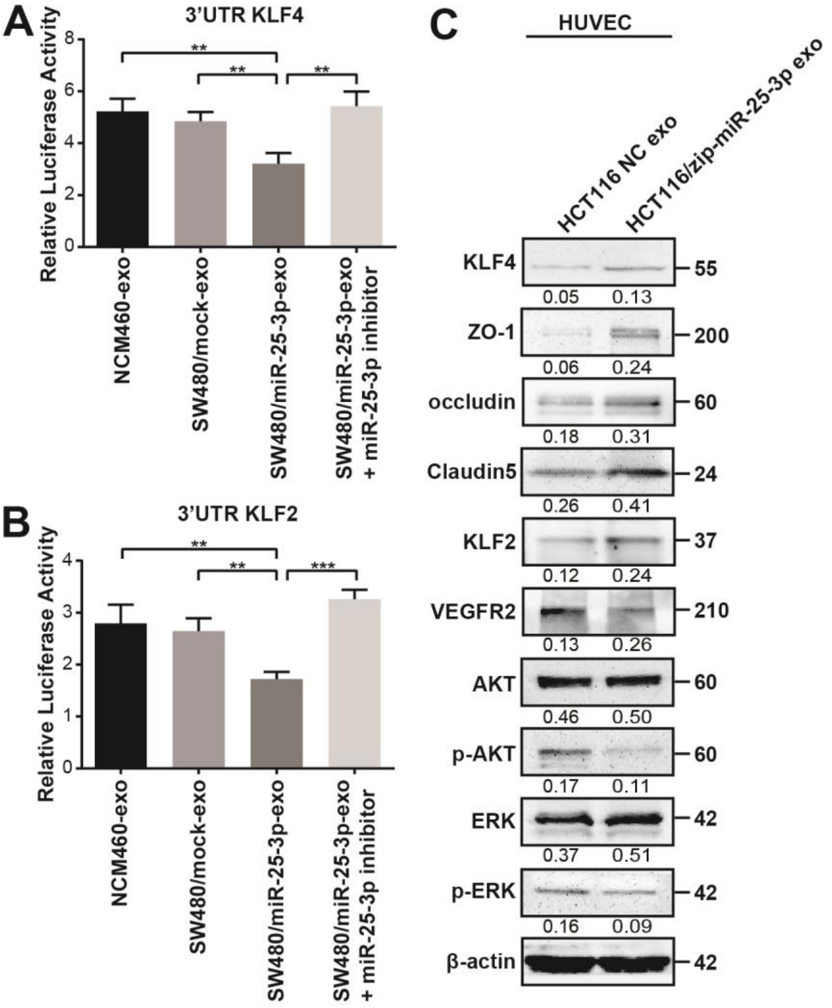

Supplementary Figure 5. Exosomal miR-25-3p hampered KLF2 and KLF4 expression. **(A and B)** Luciferase activities of 3'UTR KLF4-luc (A) and 3'UTR KLF2-luc (B) constructs in HUVECs after adding NCM460 exosomes, SW480/mock exosomes, SW480/miR-25-3p exosomes and SW480/miR-25-3p exosomes + miR-25-3p inhibitor. Mean ± s.e.m are provided (n = 3). **(C)** KLF4, ZO-1, occludin, claudin 5, KLF2, VEGFR2, AKT, p-AKT, ERK and p-ERK expression in HUVECs incubated with HCT116 NC exosomes and HCT116/zip-miR-25-3p exosomes by Western blot. **P<.01, ***P<.001 according to two-tailed Student's t test.

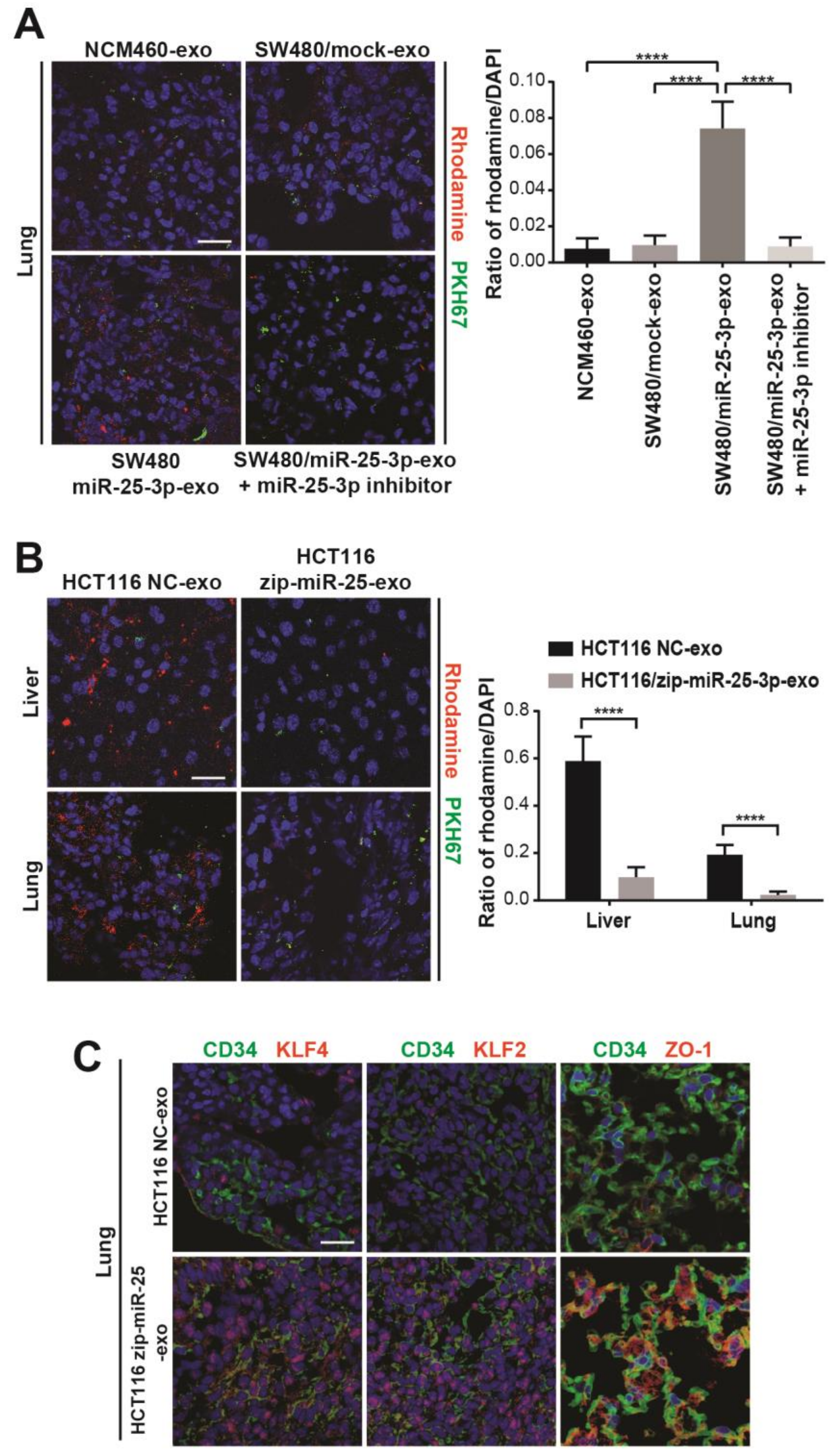

Supplementary Figure 6. CRC-secreted miR-25-3p primes vascular permeability in vivo. **(A)** Effect of NCM460 exosomes, SW480/mock exosomes, SW480/miR-25-3p exosomes, SW480/miR-25-3p exosomes + miR-25-3p inhibitor treatments on vascular permeability of mice lung by in vivo

permeability assay. The mice were injected with rhodamine-dextran after exposure to PKH67-labeled exosomes. Levels of rhodamine-dextran fluorescence in tissues were quantified using Image J software and normalized to the levels of DAPI. Mean ± s.e.m are provided (n = 5). Scale bar represents 50 µm. Scale bar represents 50 µm. **(B)** Effect of HCT116 NC exosomes and HCT116/zip-miR-25-3p exosomes on vascular permeability of mice liver and lung by in vivo permeability assay. The mice were injected with rhodamine-dextran after exposure to PKH67-labeled exosomes. Levels of rhodamine-dextran fluorescence in tissues were quantified using Image J software and normalized to the levels of DAPI. Mean ± s.e.m are provided (n = 5). Scale bar represents 50 µm. Scale bar represents 50 µm. **(C)** Effect of HCT116 NC exosomes and HCT116/zip-miR-25-3p exosomes treatments on vascular KLF4, KLF2 and ZO-1 expression (red) of mice lung by immunofluorescence. The vascular structures were labeled by CD34 (green). Scale bar represents 50 µm. ****P <.0001 according to two-tailed Student’s t test.

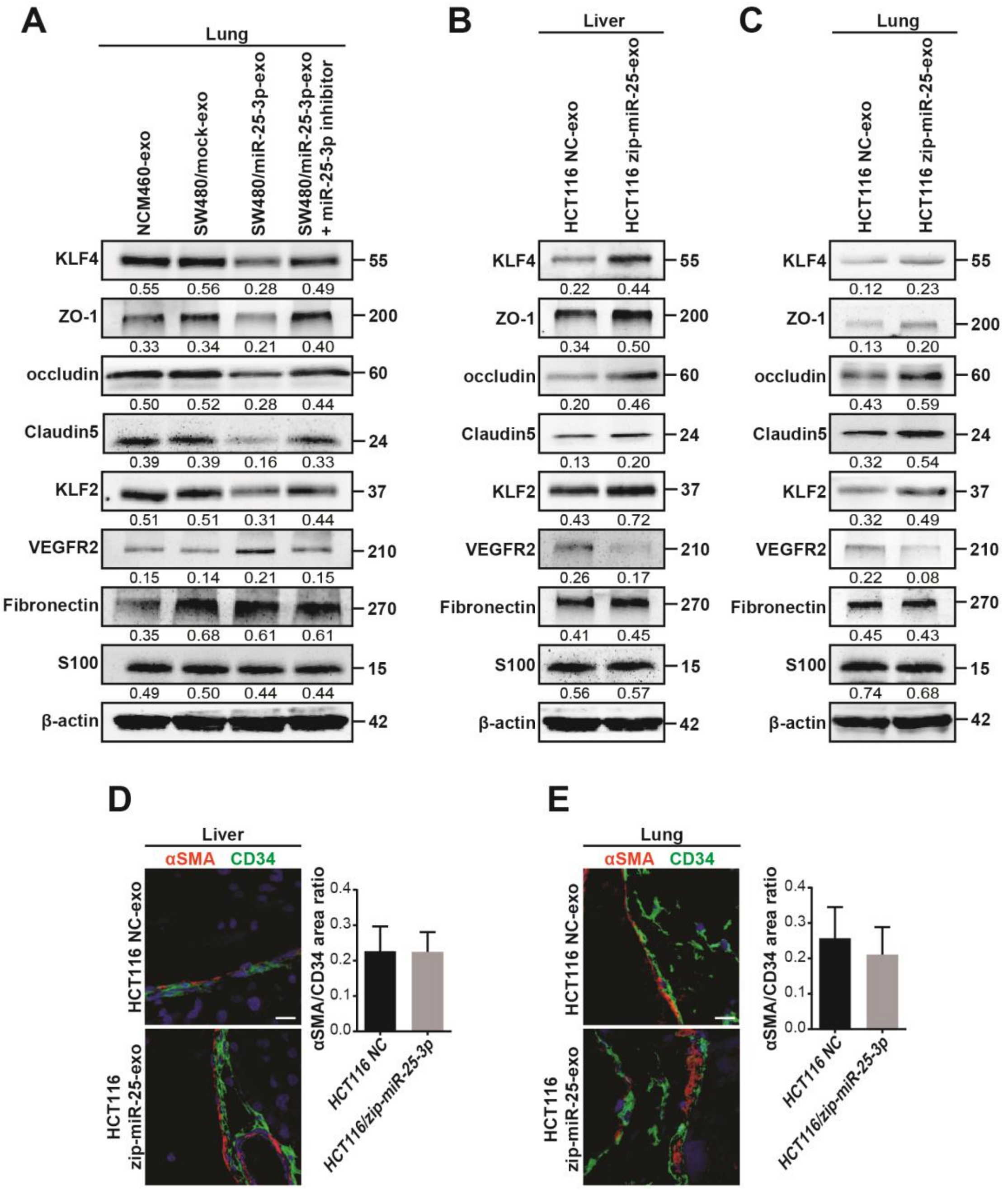

Supplementary Figure 7. The role of CRC-secreted miR-25-3p in vivo. **(A)** Effect of NCM460 exosomes, SW480/mock exosomes, SW480/miR-25-3p exosomes or SW480/miR-25-3p exosomes + miR-25-3p inhibitor treatments on KLF4, ZO-1, occludin, Claudin5, KLF2, VEGFR2, Fibronectin and S100 expression in mice lung by Western blot. **(B and C)** Effect of HCT116 NC exosomes and HCT116/zip-miR-25-3p exosomes treatments on KLF4, ZO-1,

occludin, Claudin5, KLF2, VEGFR2, Fibronectin and S100 expression in mice liver (B) and lung (C) by Western blot. **(D and E)** Effect of HCT116 NC exosomes and HCT116/zip-miR-25-3p exosomes treatments on pericyte coverage in mice liver (D) and lung (E). Pericyte coverage was quantified using Image J to measure the percentage area of αSMA+ pericytes (red) versus CD31+ vessels (green). Mean ± s.e.m are provided (n = 5). Scale bar represents 50 µm.

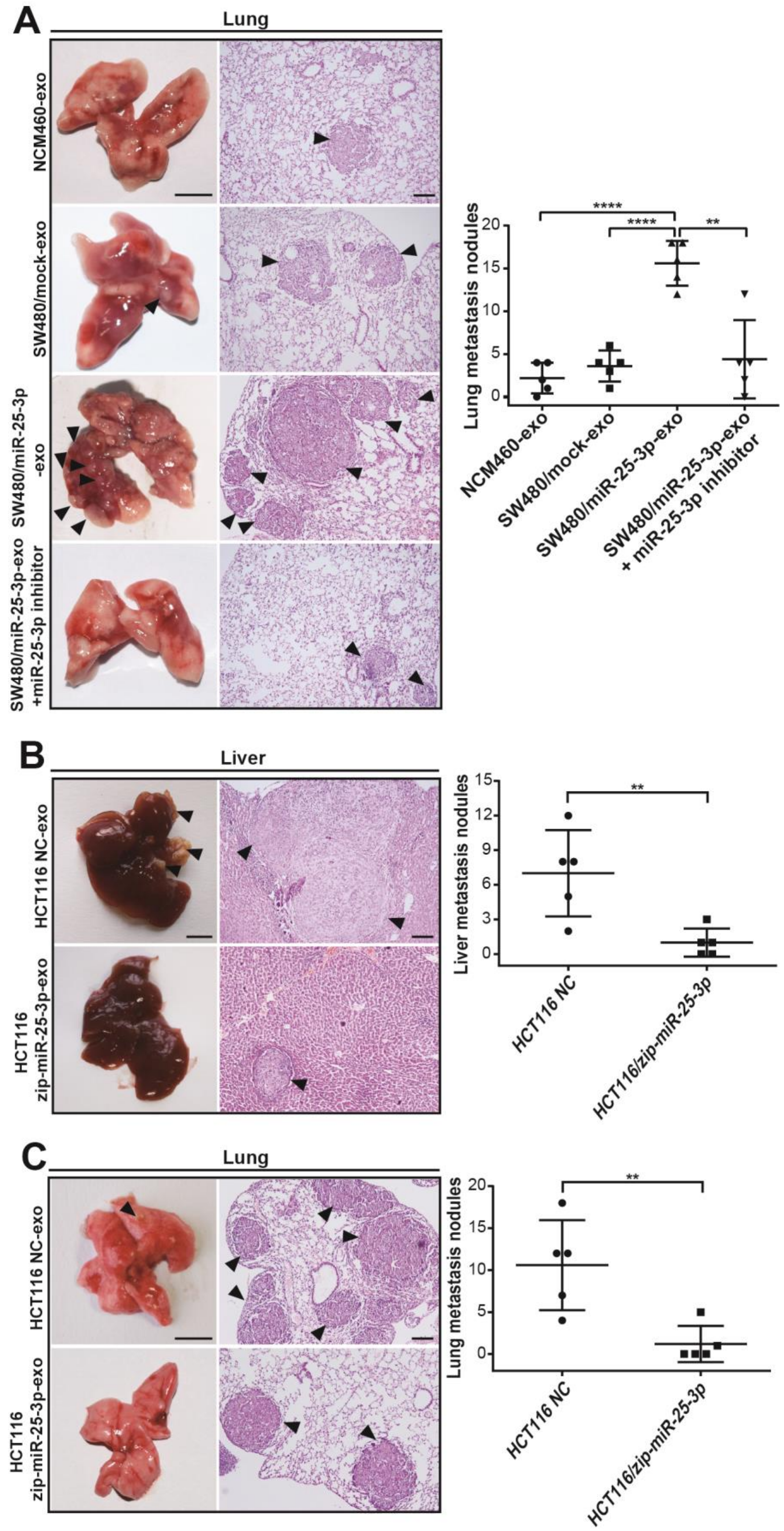

Supplementary Figure 8. CRC-secreted miR-25-3p promotes CRC

metastasis. **(A)** The mice were injected with naked SW480 cells via tail vein after exposure to NCM460 exosomes, SW480/mock exosomes, SW480/miR-25-3p exosomes or SW480/miR-25-3p exosomes + miR-25-3p inhibitor treatments. The number of lung metastatic sites (indicated by arrows) was counted under the microscope. Mean ± s.e.m are provided (n = 5). Scale bar in left panels represents 0.5 cm. Scale bar in right panel represents 100 μm. **(B and C)** The mice were injected with naked SW480 cells via tail vein or spleen after exposure to HCT116 NC exosomes and HCT116/zip-miR-25-3p exosomes treatments. The number of liver (B) and lung (C) metastatic sites (indicated by arrows) was counted under the microscope. Mean ± s.e.m are provided (n = 5). Scale bar in left panels represents 0.5 cm. Scale bar in right panel represents 100 μm. **P < .01, ****P < .0001 according to two-tailed Student's t test.

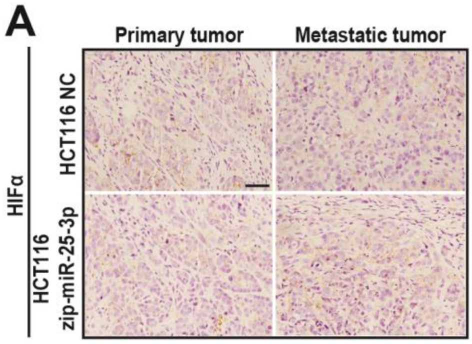

Supplementary Figure 9. MiR-25-3p has no effect on HIFα expression in CRC. **(A)** Effect of miR-25-3p knockdown on HIFα expression in primary and metastatic tumor. Scale bar represents 50 µm.

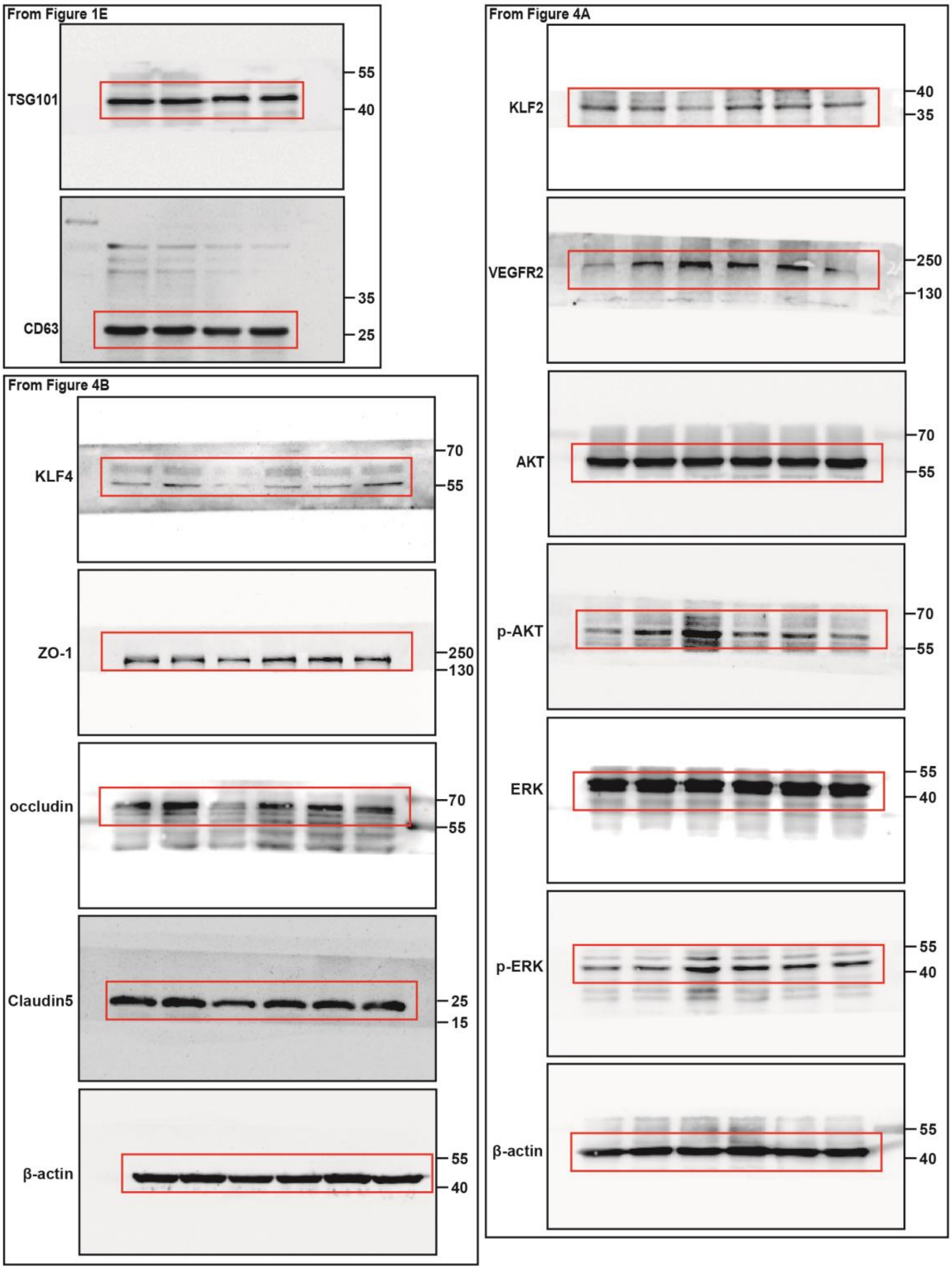

Supplementary Figure 10. Uncropped western blots from main figures. Shown are uncropped blots from figure 1E, 4A and 4B. The cropped region is highlighted with the red boxes.

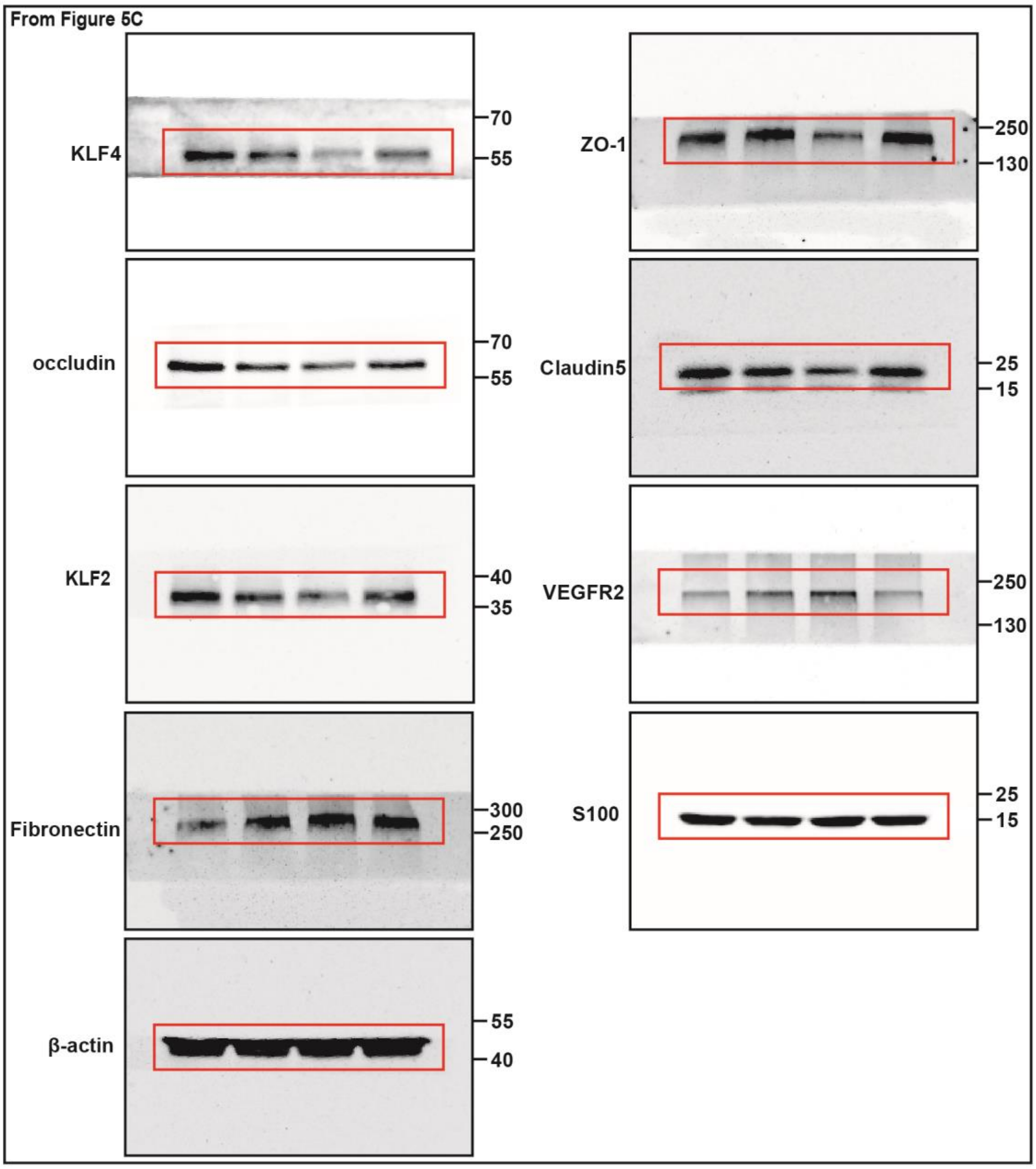

Supplementary Figure 11. Uncropped western blots from main figures. Shown are uncropped blots from figure 5C. The cropped region is highlighted with the red boxes.

Supplementary Table 1. Sequences of synthetic miRNA mimics.

Primer	Sequence(5'→3')
miR-25-3p sence	CAUUGCACUUGUCUCGGUCUGA
miR-25-3p anti-sence	AGACCGAGACAAGUGCAAUGUU
mimics-nc sense	UUCUCCGAACGUGUCACGUTT
mimics-nc anti-sense	ACGUGACACGUUCGGAGAATT

Supplementary Table 2. Sequences of synthetic miRNA inhibitor.

Primer	Sequence(5'→3')
miR-25-3p	UCAGACCGAGACAAGUGCAAUG
Inhibitor-nc	CAGUACUUUUGUGUAGUACAA

Supplementary Table 3. Primer sequences for qPCR.

**Primer**	**Sequence(5'→3')**
hsa-miR-25-3p	TTGCACTTGTCTCGGTCTGA
hsa-miR-92a	TGCACTTGTCCCGGCCTGT
hsa-miR-92b	GCACTCGTCCCGGCCTCC
hsa-miR-221	AGCUACAUUGUCUGCUGGGUUUC
hsa-miR-371-5p	AGTGCCCCCACAGTTTGAGT
hsa-miR-1246	AATGGATTTTTGGAGCAGGAA
hsa-miR-106b	TAAAGTGCTGACAGTGCAGAT
hsa-miR-93	CCCAAAGTGCTGTTCGTGC
U6-F	GGAACGATACAGAGAAGATTAGC
U6-R	TGGAACGCTTCACGAATTTGCG
pri-miR-25-F	TGTTGAGAGGCGGAGACTTG
pri-miR-25-R	GCACTGTCAGACCGAGACAA
β-actin-F	CATGTACGTTGCTATCCAGGC
β-actin-R	CTCCTTAATGTCACGCACGAT
